# Entropy Engineering Constrain Phase Transitions Enable Ultralong‐life Prussian Blue Analogs Cathodes

**DOI:** 10.1002/advs.202402340

**Published:** 2024-04-26

**Authors:** Yuhao Lei, Shiyong Wang, Lin Zhao, Changping Li, Gang Wang, Jieshan Qiu

**Affiliations:** ^1^ School of Environment and Civil Engineering Research Center for Eco‐environmental Engineering Dongguan University of Technology Dongguan Guangdong 523106 P. R. China; ^2^ College of Chemical Engineering Beijing University of Chemical Technology Beijing 100029 P. R. China

**Keywords:** capacitive deionization, entropy engineering, high‐entropy materials, prussian blue analogs, ultralong‐life

## Abstract

Prussian blue analogs (PBAs) are considered as one of the most potential electrode materials in capacitive deionization (CDI) due to their unique 3D framework structure. However, their practical applications suffer from low desalination capacity and poor cyclic stability. Here, an entropy engineering strategy is proposed that incorporates high‐entropy (HE) concept into PBAs to address the unfavorable multistage phase transitions during CDI desalination. By introducing five or more metals, which share N coordination site, high‐entropy hexacyanoferrate (HE‐HCF) is constructed, thereby increasing the configurational entropy of the system to above 1.5R and placing it into the high‐entropy category. As a result, the developed HE‐HCF demonstrates remarkable cycling performance, with a capacity retention rate of over 97% after undergoing 350 ultralong‐life cycles of adsorption/desorption. Additionally, it exhibits a high desalination capacity of 77.24 mg g^−1^ at 1.2 V. Structural characterization and theoretical calculation reveal that high configurational entropy not only helps to restrain phase transition and strengthen structural stability, but also optimizes Na^+^ ions diffusion path and energy barrier, accelerates reaction kinetics and thus improves performance. This research introduces a new approach for designing electrodes with high performance, low cost, and long‐lasting durability for capacitive deionization applications.

## Graphical abstract

The HE‐HCF electrode, which belongs to the high‐entropy category, exhibits excellent desalination performance, including an impressive salt adsorption capacity and its remarkable cyclic stability. Structural characterization and DFT studies show that HE‐HCF exhibits unprecedented enhancement in desalination performance due to its high entropy effect, low Na^+^ migration energy barrier, excellent reaction reversibility and, remarkable structural stability.

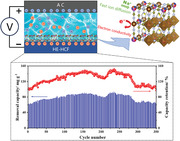

## 1 Introduction

In recent years, the problem of environmental pollution has become more and more prominent, and the shortage of water resources has become a global crisis, the conversion of seawater and brackish water into freshwater is an effective way to solve the freshwater crisis.^[^ ^1^ ^]^ Capacitive deionization (CDI) has received much attention from researchers within the environmental field as an emerging efficient and green water treatment technology, and is being considered as a low‐energy, economical alternative to conventional water treatment technologies such as reverse osmosis and electrodialysis.^[^ ^2^ ^]^ As a branch of the electrochemical field, CDI, like most electrochemical reactions in that the electrode material is of paramount importance to achieve efficient and rapid desalination.^[^ ^3^ ^]^ Over the past decade, four main materials have been extensively studied as cathode materials for desalination, including Prussian blue analogs (PBAs),^[^ ^4^ ^]^ transition metal oxides,^[^ ^5^ ^]^ sulfides,^[^ ^6^ ^]^ and 2D materials such as Mxene.^[^ ^7^ ^]^ Among them, PBAs are a group of coordination compounds with a general formula of A*_x_* M_1_[M_2_(CN)_6_]*_y_*_□_ *_m_* ·*_n_* H_2_O (where A is usually alkali ions, M_1_/M_2_ are transition metals (with M_2_ typically Fe), and □ denotes the [M_2_(CN)_6_] vacancies (0 < *x* < 2, 0 < *y* < 1, *y* + *m* = 1).^[^ ^8^ ^]^ They are considered as one of the most promising cathodes for electrochemical desalination due to their low cost, excellent theoretical sodium storage capacitance, and 3D open framework for fast Na^+^ transport.^[^ ^9^ ^]^ However, the inherent low conductivity and poor stability of PBAs limit their application in desalination, and charge‐transfer materials usually fail to balance both high capacity and good stability.^[^ ^10^ ^]^ Theoretically, higher capacity means that more ions are removed, but this usually leads to more pronounced volume changes.^[^ ^11^ ^]^ If the material fails to mitigate the phase transition that accompanies the reaction, the performance will also degrade irreversibly. Therefore, designing an electrode material with good stability and high reliability is crucial for desalination applications.

Incorporating high‐entropy (HE) concept into PBAs may help solve the above problems. Yeh et al. listed four factors that affect the properties of high‐entropy materials, namely configuration entropy, sluggish diffusion, cocktail effect, and lattice distortion, which can improve the capacity and cycling performance of electrode materials.^[^ ^12^ ^]^ As an emerging concept in the materials field in recent years, it can help stabilize the cathode by maximizing the configurational entropy of the structure.^[^ ^13^ ^]^ In the last few years, various high‐entropy materials (HEMs), i.e., five or more metal species sharing the same lattice positions (compounds containing more than five elements are usually defined as HE compounds) have been combined into single phases for a wide variety of applications, including catalysis,^[^ ^14^ ^]^ chemical^[^ ^15^ ^]^ and energy storage,^[^ ^16^ ^]^ most of which are high‐entropy oxides (HEOs)^[^ ^17^ ^]^ and high‐entropy alloys (HEAs).^[^ ^18^ ^]^ HEMs can be used for electrochemical energy storage because they exhibit properties beyond those of their constituent elements, while their unique structure provides excellent properties unmatched by conventional materials with only one or two major elements.^[^ ^19^ ^]^

Although PBAs have not yet attracted much attention in the field of high‐entropy materials, it has recently been shown that the M active sites in PBAs can be replaced by other redox transition metals, such as Cr, Mn, Co, Ni, Cu, etc., which can change their electrochemical behavior.^[^ ^20^ ^]^ It is well known that stability is an important characteristic of desalination electrodes, and high stability means longer desalination cycle and longer service life,^[^ ^21^ ^]^ and the introduction of high‐entropy concept can solve the problems of poor conductivity and unstable performance of PBAs. Meanwhile, the increase of the configurational entropy and the large entropy grouping can stabilize the crystal structure of PBAs, which makes it more difficult for the 3D crystal structure of PBAs to collapse, thus improving the stability of desalination.^[^ ^22^ ^]^

In this paper, based on the entropy engineering strategy, a high‐entropy hexacyanoferrate (HE‐HCF) of five metals sharing N coordination was prepared by simple co‐precipitation method, and it was used as the CDI cathode material for the first time to solve the problems of low desalination capacity and poor cycling performance caused by the adverse phase transition of PBAs in the CDI process. Similarly, the medium‐entropy HCF with the M site containing three or four transition metal elements were named ME‐HCFs, and the low‐entropy HCF with the M site containing only one or two transition metal elements were named LE‐HCFs. We compare the physicochemical properties and electrochemical desalination behaviors of HE‐HCF with those of ME and LE‐HCFs as well as some of the novel Faradaic electrodes, the results show that HE‐HCF has a very impressive long cycle life and desalination capacity, which is attributed to the high configurational entropy that effectively improves the sodium storage capacity and structural framework strength. Meanwhile, the increased configurational entropy successfully suppressed the unfavorable phase transition and accelerated the ion diffusion during the cycling process, which solved the problems of poor reversibility and capacity decay. This provides multiple possibilities for advancing the entropy engineering strategy for a wide range of applications.

## 2 Results and Discussion

**Figure 1:**
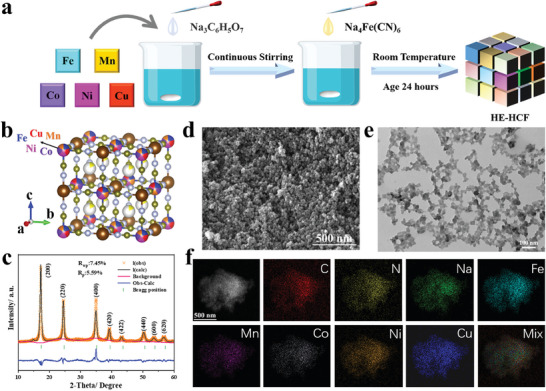
a) Schematic illustration of the preparation procedure of the HE‐HCF. b) The ball‐and‐stick model of HE‐HCF. c) Neutron powder diffraction Rietveld refinement pattern of the HE‐HCF sample. d,e) SEM and TEM images of HE‐HCF. f) STEM image and the corresponding elemental maps.

The HE‐HCF was prepared by co‐precipitation method, and other materials with different configurations of entropy can be prepared by sequentially reducing the types of transition metal elements added, with the schematic illustration shown in **Figure** **1**a. The structure of HE‐HCF is described in Figure 1b, with five equimolar transition metal elements Fe, Mn, Co, Ni, and Cu sharing the nitrogen‐coordinated M sites (0.2 mole fraction of each element) in the PBAs lattice (Na_X_M[Fe(CN)_6_]), as determined using the statistical thermodynamics‐derived definition of ΔS_conf_, see Equations S1 and S2 (Supporting Information), the configurational entropy (ΔS_conf_) of HE‐HCF was calculated to be 1.61R. The ΔS_conf_ values of ME‐HCFs and LE‐HCFs are also different depending on the number of elements. The ΔS_conf_ of quaternary, ternary, binary, and monovalent PBAs are 1.39R, 1.10R, 0.69R, and 0R, respectively. X‐ray diffraction (XRD) patterns of all samples showed the typical cubic crystal structure of the Fm‐3m space group (JCPDS 73–0687) and no impurities were observed (Figure S1a, Supporting Information).^[^ ^23^ ^]^ Then, the refinement was performed by the Rietveld method (weighted contour R factor, *R*_wp_ = 7.45% and contour R factor *R*_p_ = 5.59%) (Figure 1c; Table S1, Supporting Information). The lattice parameters of HE‐HCF are measured to be a = b = c = 10.25012Å, α = β = γ = 90.0000°, and V = 1076.929Å^3^. Thermogravimetric analysis (TGA) exhibits that the weight loss of HE‐HCF at 170 and 270 °C is 15.7% and 24.4% (Figure S1b, Supporting Information), respectively. Combining Inductively Coupled Plasma Emission Spectroscopy (ICP‐OES) and Elemental Analysis (EA) Results (Tables S2 and S3, Supporting Information), the chemical formula of HE‐HCF was estimated to be Na_1.117_Fe_0.182_Mn_0.216_Co_0.183_Ni_0.219_Cu_0.2_[Fe(CN)_6_]_0.768_o_0.232_·2.15H_2_O, while the chemical formulas of all samples are listed in the Table S4 (Supporting Information). Fourier transform infrared spectroscopy (FTIR) and Raman spectroscopy were used to explore the coordination environment of chemical bonds in five samples and characterize their structures. The prominent peaks observed at 2101 and 2134.1 cm^−1^ in the Raman spectroscopy, along with the broad peak detected at 2095.2 cm^−1^ in the FTIR spectrum, can be attributed to the presence of C≡N groups coordinated to the transition metal (Figure S1c,d, Supporting Information).^[^ ^24^ ^]^ The vibrational bands of the FTIR absorption peaks are located at 1617.9 and 3438.1 cm^−1^, respectively, due to the presence of H─O─H and H─O bending modes of the H_2_O molecules.^[^ ^23a^ ^]^ According to SEM and TEM, the morphology of HE‐HCF and other samples are fine particles with smooth surface (Figure 1d,e; Figures S2 and S3, Supporting Information). The energy dispersive X‐ray energy spectrum (EDS) of the HE‐HCF (Figure 1f) confirms the uniform distribution of Fe, Mn, Co, Ni, Cu, C, N, and Na. The same information was obtained for EDS of other samples (Figures S4–S7, Supporting Information), indicating that the coprecipitation method is suitable for the preparation of HCFs containing single or multiple transition metals.

**Figure 2:**
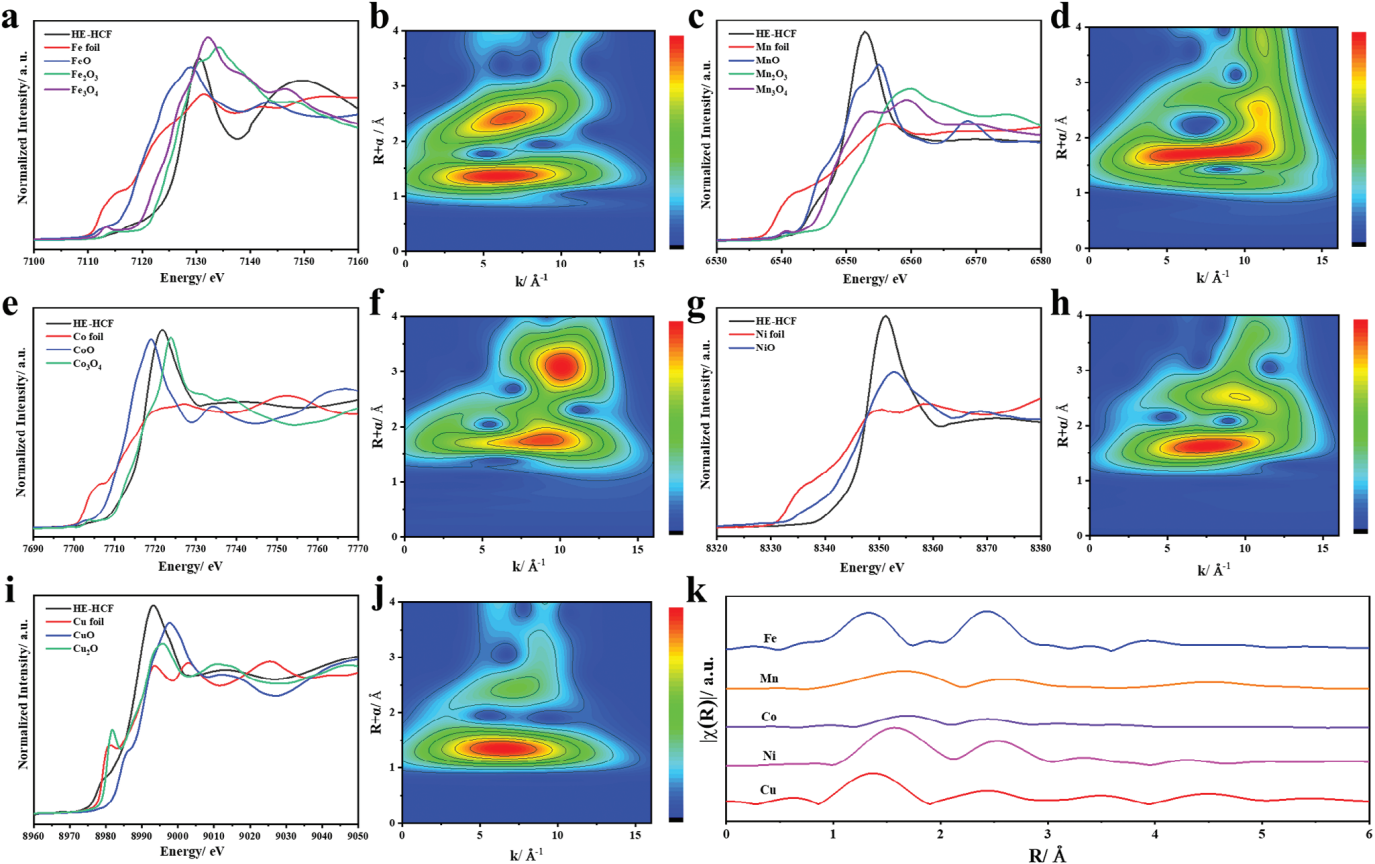
a) Fe K‐edge XANES spectra of the transition metals in HE‐HCF. b) Wavelet transforms (WT) of Fe sample in HE‐HCF. c) Mn K‐edge XANES spectra of the transition metals in HE‐HCF. d) Wavelet transforms (WT) of Mn sample in HE‐HCF. e) Co K‐edge XANES spectra of the transition metals in HE‐HCF. f) Wavelet transforms (WT) of Co sample in HE‐HCF. g) Ni K‐edge XANES spectra of the transition metals in HE‐HCF. h) Wavelet transforms (WT) of Ni sample in HE‐HCF. i) Cu K‐edge XANES spectra of the transition metals in HE‐HCF. j) Wavelet transforms (WT) of Cu sample in HE‐HCF. k) Fourier transform of the EXAFS signals collected from the HE‐HCF.

The local coordination environments and valence states of different metal ions have been studied by X‐ray absorption spectroscopy (XAS). **Figure** **2**a–j display the normalized K‐edge X‐ray absorption near edge spectroscopy (XANES) of Fe, Mn, Co, Ni, and Cu, as well as the corresponding wavelet transform (WT), showing different edge positions close to FeO, MnO, CoO, NiO, and CuO, respectively, indicating that the stable valence states of these five transition metal elements are ≈+2. The K‐side extended X‐ray absorption fine structure (EXAFS) spectra of Fe, Mn, Co, Ni, and Cu in HE‐HCF display significant Fe─N, Mn─N, Co─N, Ni─N, and Cu─N, this further indicates that five transition metal elements, Fe, Mn, Co, Ni, and Cu, were successfully introduced into the framework of HE‐HCF (Figures S8 and S9, Supporting Information) and the WT corresponding to the oxidation states of different metal ions are illustrated in Figures S10–S14 (Supporting Information). The corresponding Fourier transform of the extended X‐ray absorption fine structure (EXAFS) signal is plotted in Figure 2k, the radial distributions are very similar, indicating a homogeneous distribution of transition metal cations at the mixed metal sites.^[^ ^22b^ ^]^ The surface composition and elemental valence states of HE‐HCF were further investigated using X‐ray photoelectron spectroscopy (XPS). As demonstrated in Figure S15a (Supporting Information), the survey XPS spectra of HE‐HCF shows the characteristic peaks of C 1s, N 1s, O 1s, Na 1s, Fe 2p, Mn 2p, Co 2p, Ni 2p, Cu 2p. Figure S15b–g (Supporting Information) demonstrate the detailed XPS spectra of transition metal elements. The Na 1s spectrum shows a single characteristic peak at binding energy of 1072.5 eV.^[^ ^25^ ^]^ The characteristic peaks of Fe 2p appearing at binding energies of 708.6 and 721.5 eV, representing Fe 2p_3/2_ and Fe 2p_1/2_ of Fe^2+^, respectively. The characteristic peaks at binding energies of 710 and 723 eV are Fe 2p_3/2_ and Fe 2p_1/2_ of Fe^3+^, respectively.^[^ ^26^ ^]^ The characteristic peaks of the Mn 2p spectrum at the binding energy positions of 642 and 653.5 eV are attributed to Mn 2p_3/2_ and Mn 2p_1/2_ of Mn^2+^.^[^ ^27^ ^]^ In the spectrum of Co 2p, in addition to the corresponding Co^2+^ peaks at 785.9 eV (Co 2p_3/2_) and 798.7 eV (Co 2p_1/2_), two different peaks representing Co 2p_3/2_ and Co 2p_1/2_ for Co^3+^ at 782.4 and 797.5 eV, respectively, as well as binding energies at 789.5 and 805.5 eV satellite peaks at binding energies of 789.5 and 805.5 eV.^[^ ^28^ ^]^ The Ni 2p spectrum exhibit major peaks at 856.4 eV (Ni 2p_3/2_) and 874.1 eV (Ni 2p_1/2_), as well as satellite peaks at binding energies 863.3 and 880.5 eV, which are typical of Ni^2+^.^[^ ^29^ ^]^ The small peaks at slightly higher binding energies 858 and 875.3 eV can be attributed to Ni^3+^.^[^ ^30^ ^]^ Similarly, in the final spectrum of Cu 2p, shake‐up satellite peaks were detected at binding energies of 944.2 eV as well as 963.6 eV, respectively, indicating the presence of Cu^2+^, the characteristic peaks of Cu^2+^ (2p_3/2_) and Cu^2+^ (2p_1/2_) corresponded to 935.6 and 955.4 eV, and two peaks with smaller areas of 933.1 and 952.8 eV appeared in the spectrum of Cu 2p, corresponding to Cu^1+^ (2p_3/2_) and Cu^1+^ (2p_1/2_), respectively.^[^ ^31^ ^]^ This demonstrates that an redox reaction from Cu^2+^ to Cu^1+^ occurred within the structure during synthesis, along with the partial oxidation of Ni^2+^ to Ni^3+^.^[^ ^32^ ^]^ Similar XPS results were obtained for all ME‐HCFs and LE‐HCFs, (Figures S16–S19, Supporting Information). According to Brunauer–Emmett–Teller (BET) surface area analysis (Figure S20, Supporting Information), the specific surface area of HE‐HCF is 176.8 m^2^ g^−1^, which is higher than the LE HCFs (Fe‐HCF is 91.7 m^2^ g^−1^, FeMn‐HCF is 98.8 m^2^ g^−1^) and ME HCFs (FeMnCo‐HCF is 74.5 m^2^ g^−1^, FeMnCoNi‐HCF is 150.9 m^2^ g^−1^). The specific surface area of the sample increases concurrently with the increase in entropy. A relatively high specific surface area is beneficial for increasing the contact area between the electrode and the salt solution, promoting rapid ion diffusion.^[^ ^33^ ^]^

**Figure 3:**
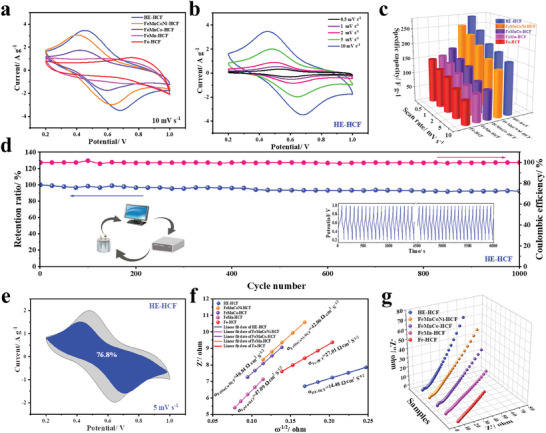
a) Cyclic voltammograms for HCFs at a scan rate of 10 mV s^−1^. b) CV curves of HE‐HCF at different scan rates. c) Specific capacitance values. d) Cycling performance and coulombic efficiency at 2 A g^−1^ for 1000 GCD cycles. e) The contribution of capacitive‐controlled (blue) and diffusion‐controlled process (grey) of HE‐HCF. f) Fitting results for Z' and ω^−1/2^ in the low frequency range. g) EIS curve.

The electrochemical behaviors of the samples were investigated in a three‐electrode system with 1 m NaCl solution as the electrolyte, aiming to emphasize the electrochemical advantages of HE‐HCF. The cyclic voltammetry (CV) curve in **Figure** **3**a shows a pair of well‐defined redox peaks at ≈0.45/0.68 V, corresponding to the reversible insertion and extraction of Na^+^ ions in PBAs.^[^ ^34^ ^]^ It is clear that the HE‐HCF displays a larger peak current density and integral area of the CV curve at a scan rate of 10 mV s^−1^ compared to the ME‐HCFs and LE‐HCFs, indicating a higher sodium storage capacity and better capacitive performance. Next, CV tests were performed on the samples at a scanning rate of 0.5–10 mV s^−1^ (Figure 3b; Figure S21, Supporting Information). The shape of the redox peaks did not change with increasing scan rate, indicating good rate performance. Due to the accessibility of ion diffusion, the specific capacitance of each sample decreases with the increase of scanning rate, but the specific capacitance of HE‐HCF is the largest at any scanning rate (Figure 3c), and even reaches the ultra‐high specific capacitance of 280.4 F g^−1^ at 0.5 mV s^−1^ (Figure S22 and Equation S3, Supporting Information). Figure S23 (Supporting Information) shows the galvanostatic charge/discharge (GCD) curve for all HCF samples at 0.1 A g^−1^ with potential window of 0.2–1.0 V. HE‐HCF has a longer discharge time than that of ME‐HCFs and LE‐HCFs, reflects the high‐entropy enhanced capacitive performance. In addition, the capacity of HE‐HCF at different current densities was also significantly better than that of ME‐HCFs and LE‐HCFs (Figure S24, Supporting Information), which fully confirms that the entropy engineering strategy can effectively improve the ion storage performance of HCFs. To study the effect of entropy on electrochemical stability, the sample was charged and discharged 1000 times at the current density of 2 A g^−1^. After 1000 cycles, the capacitance retention rate of HE‐HCF is 92.3%, and the average coulomb efficiency is close to 100%, which is significantly better than other ME‐HCFs and LE‐HCFs (Figure 3d; Figure S25, Supporting Information). When comparing the electrochemical performance with traditional Fe‐HCF, the advantages are even more pronounced, as the Fe‐HCF material exhibits disappointing result in continuous cycling, with only ≈30% retention after 1000 cycles, indicating that altering the ΔS_conf_ of the system has a significant impact on cycle stability. According to the color comparison of the electrolyte after GCD cycling, it can be seen that with the decrease of the configurational entropy, the stability of different materials themselves deteriorate to the point that the materials precipitate from the electrode into the electrolyte during the cycling process resulting in different degrees of discoloration of the NaCl solution, which most maintains a colorless and transparent electrolyte after the cycling of HE‐HCF to prove its amazing stability (Figure S26, Supporting Information).

The relationship between the peak current (i) and scanning rate (v) of the electrode material at different voltages was studied by using the power law formula (Equation S4, Supporting Information) to explore the electrochemical storage dynamics.^[^ ^35^ ^]^ According to the fitting results (Figure S27, Supporting Information), HE‐HCF, FeMnCoNi‐HCF, FeMnCo‐HCF, FeMn‐HCF, and Fe‐HCF are 0.81, 0.79, 0.84, 0.94, 0.82 (anode) and 0.80, 0.80, 0.73, 0.76, 0.73 (cathode), respectively. This means that the combination of ion diffusion and capacitive behavior synergistically controls the charge storage process. And we calculated the current contribution of capacitive control and diffusion control processes for each HCF electrode using the Dunn method (Equation S5, Supporting Information).^[^ ^36^ ^]^ The capacitive contribution of HE‐HCF reaches 76.8% at 5 mV s^−1^ (Figure 3e), which is higher than that of FeMnCoNi‐HCF (75.1%), FeMnCo‐HCF (74.5%), FeMn‐HCF (71.5%), and Fe‐HCF (64.2%) (Figure S28, Supporting Information). The capacitive contribution of each HCF material is positively correlated with the scanning rate (Figure S29, Supporting Information), and the capacitive contribution of HE‐HCF is up to 85.5% at a scanning rate of 10 mV s^−1^. By comparison, at different sweep speeds, the capacitive contribution of the HE‐HCF is greater than that of the ME‐HCFs and LE‐HCFs, and as the configurational entropy decreases, the proportion of the capacitive contribution also decreases. The maximum capacitive contribution is conducive to fast and reversible sodium ion reaction kinetics, while ensuring the high‐rate capability of the HE‐HCF electrode.^[^ ^37^ ^]^ Electrochemical impedance spectroscopy (EIS) of the electrode material was used to analyze the charge transfer efficiency. Figure S30 (Supporting Information) shows the low‐frequency and magnified high‐frequency regions of the Nyquist plot. Compared with several other HCF electrodes, the HE‐HCF exhibits the smallest semicircular diameter, meaning the smallest charge transfer resistance (*R*_ct_). The Warburg coefficient (σ*_w_*) obtained by plotting the relationship curve between Z' and *w^−^* ^1/2^ (*w* is the angular frequency) corresponds to the Na^+^ ions diffusion rate of the electrode in the low frequency range.^[^ ^38^ ^]^ As can be seen in Figure 3f, the HE‐HCF has the smallest slope, indicating that the diffusion rate of Na^+^ ions is faster than the other four HCF electrodes, which is more conducive to rapid sodium removal. In addition, the 3D view of Figure 3g provides a more intuitive indication that the linear curve of HE‐HCF has steeper line gradients and larger slopes. It is evident that HE‐HCF clearly has a faster charge/ion transfer rate.

**Figure 4:**
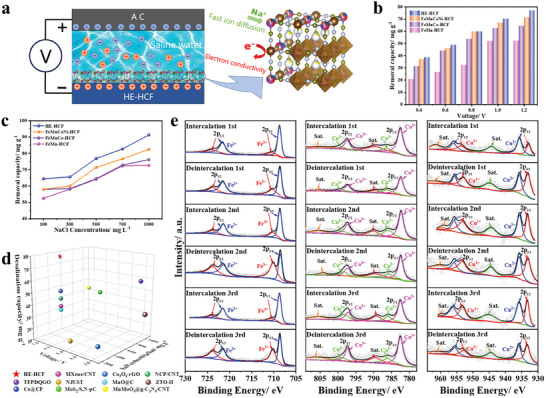
a) Schematic diagram of desalination with HCDI architecture. b) The desalination capacity of HCFs in 500 mg L^−1^ NaCl solution at various voltage. c) The desalination capacity of HCFs at various concentrations NaCl solution at 1.2 V. d) Comparison of desalination performances of the HE‐HCF and those based on other reported Faradaic electrode materials. e) The XPS spectra of Fe 2p, Co 2p and Cu 2p during Na^+^ intercalation and deintercalation in 1st, 2nd, and 3rd cycles.

To verify the desalination performance, we constructed a HCDI module, where HE‐HCF and AC were used as cathode and anode, respectively (**Figure** **4**a). During the desalination process, Na^+^ ions are inserted into the HE‐HCF electrode, while Cl^−^ ions are captured by the AC electrode.^[^ ^39^ ^]^ Figure 4b and Figure S31 (Supporting Information) demonstrate the desalination capacity and corresponding conductivity change curves of different HCF under different voltage conditions when the initial concentration of NaCl solution is 500 mg L^−1^ (Equation S6, Supporting Information). HE‐HCF exhibits an ultra‐high desalination capacity of 77.24 mg g^−1^ at a voltage of 1.2 V, which is higher than the other three materials, FeMnCoNi‐HCF (74.44 mg g^−1^), FeMnCo‐HCF (64.5 mg g^−1^), and FeMn‐HCF (52.3 mg g^−1^). Traditional EDL based capacitive deionization is not compatible with high salt concentration water due to its co‐ionic efflux effect, which can easily lead to the loss of effectiveness of the material.^[^ ^40^ ^]^ Therefore, maintaining a high desalination capacity at various salt concentrations is crucial. We investigated the CDI performance of each sample in 100–1000 mg L^−1^ NaCl solution. With the review of salt solubility, the desalination capacity gradually increased, and the maximum desalination capacity of HE‐HCF in 1000 mg L^−1^ NaCl solution at 1.2 V was 91.24 mg g^−1^, which was significantly higher than the other three materials (Figure 4c). This result once again verifies that the attraction of HCFs to Na^+^ increases with increasing ΔS_conf_, and the high configurational entropy significantly improves the reversible sodium storage performance of this system. It should be noted that because the stability of Fe‐HCF is so poor that very serious discoloration occurs as soon as the salt solution passes through the electrode, it is not tested in the desalination capacity and the following cycling capacity investigation (Figure S32, Supporting Information). Compared with the recently reported Faradaic electrode, the results of HE‐HCF in desalination capacity are satisfactory (Figure 4d).^[^ ^41^ ^]^

To better understand the role of each transition metal element in HE‐HCF, the first three CDI cycles of HE‐HCF were performed, and the samples after each stage were separately XPS analyzed (Figure 4e; Figure S33, Supporting Information). For the pristine samples, the transition metal elements such as Fe, Mn, Co, Ni, and Cu mainly remain in the divalent valence state. Since the presence of original Na in the HCF framework will inevitably affect the first adsorption/desorption process, the XPS spectra of the second and third cycles are mainly analyzed. The Fe 2p spectra showed that part of the Fe^3+^ in the electrode was reduced to Fe^2+^ after adsorption in each CDI cycle. In the last two cycles, compared with the XPS spectrum after the previous cycle, the proportion of characteristic peak areas of Fe^2+^ in the adsorption stage increased, while the proportion of characteristic peak areas of Fe^3+^ decreased, which indicated that with the insertion of Na^+^ ions, it was accompanied by the conversion of Fe^3+^ to Fe^2+^. After desorption, the proportions of the characteristic peaks of Fe^2+^ and Fe^3+^ showed an opposite trend, and the characteristic peak areas of Fe^2+^ and Fe^3+^ decreased and increased, respectively. Meanwhile, Co^2+^/Co^3+^ and Cu^1+^/Cu^2+^ showed the same trend as Fe^2+^/Fe^3+^, where Co^3+^ and Cu^2+^ were partially reduced to Co^2+^ and Cu^1+^ during the adsorption process, while the opposite was observed during the desorption process. In the adsorption phase of the last two cycles, the proportion of the characteristic peaks of Cu^1+^ and Co^2+^ increased and the proportion of the characteristic peak areas of Cu^2+^ and Co^3+^ decreased, while the desorption process showed the opposite trend to the adsorption process. The valence states of the above elements have similar trends, indicating that they are involved in reversible redox reactions. In comparison, the chemical valences and ratios of Mn 2p and Ni 2p did not change significantly, indicating that Mn and Ni exhibit redox inertia. Based on the above discussion, we conclude that Fe^2+^/^3+^, Co^2+/3+^ and Cu^1+^/^2+^ are the major redox centers in HE‐HCF.

**Figure 5:**
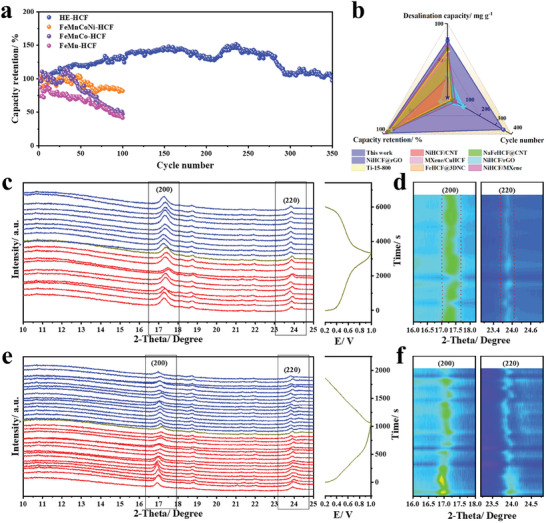
a) Cyclic desalination performances of HCFs at 1 V. b) Comparison of the desalination and cycle performance of HE‐HCF film with other HCFs electrode materials. c) The ex situ XRD analysis with the corresponding charge–discharge curve from HE‐HCF. d) The contour plots of XRD patterns for the HE‐HCF. e) The ex situ XRD analysis with the corresponding charge‐discharge curve from FeMn‐HCF. f) The contour plots of XRD patterns for the FeMn‐HCF.

As a key point that cannot be ignored as a CDI electrode, the cycle performance is very important for practical promotion. As shown in **Figure** **5**a, the performance of HE‐HCF was not significantly degraded after 350 cycles in 500 mg L^−1^ NaCl solution with 1 V voltage applied, with capacity retention above 97%, which demonstrating the HE‐HCF has excellent cycling stability. To our knowledge, this is the best cycling performance of PBAs materials reported to date. The desalination capacity of HE‐HCF increased and stabilized over the first dozens of cycles, which may be attributed to the increased activation and hydrophilicity of HE‐HCF.^[^ ^42^ ^]^ In contrast, the cycling curves of the other three samples showed varying degrees of capacity degradation after only 100 revolutions, suggesting that serious side reactions were occurring within the electrode, which affected the adsorption performance of the electrode. The ions of the transition metal elements in the HE‐HCF were almost not detected in the NaCl solution after cycling, and the negative correlation with the magnitude of capacity retention is that the Fe and Mn ions in the solution after cycling of the FeMn‐HCF are the most abundant, even to the extent that the NaCl solution is obviously discolored (Figure S34, Table S5, Supporting Information). This indicates that the use of HE‐HCF as CDI electrode do not cause secondary contamination of the solution, while ME‐HCFs and LE‐HCFs cause different degrees of contamination of the solution. This further confirms that ΔS_conf_ has a positive effect on the reversibility of the reaction. Meanwhile, according to the XRD patterns of all HCFs before and after cycling, the HE‐HCF shows no significant change in the diffraction peaks after repeated insertion and extraction of Na^+^ ions, and the post‐cycling XRD characteristics are highly coexisting with those of the pre‐cycling HCFs, whereas the electrodes of the ME‐HCFs and the FeMn‐HCF show new diffraction peaks at different locations (Figure S35, Supporting Information). Scanning and transmission electron microscopy (SEM and TEM) were carried out on the electrodes after circulation, and it was found that HE‐HCF was still in granular state, and no obvious morphological changes were observed on the surface (Figures S36 and S37, Supporting Information). Relatively, as the ΔS_conf_ decreases, the electrode particles of ME‐HCFs and FeMn‐HCF after cycling show different degrees of deformation, among which FeMn‐HCF exhibits obvious fragmentation, which may be responsible for its poor cycling performance. The XPS analysis of the HCFs after the cycle display that the characteristic peak positions of each transition metal element do not shift significantly after the adsorption‐desorption cycle (Figures S38–S41, Supporting Information). Comparing the performance of HE‐HCF with other recently reported Prussian blue base CDI electrodes, the combined strength of HE‐HCF is far ahead in terms of desalinization capability and cycle stability (Figure 5b; Table S6, Supporting Information).^[^ ^7^, ^43^ ^]^ From all the above results, it can be observed that HE‐HCF has high desalination capacity and excellent cycling stability, which lays a solid foundation for its application in seawater desalination.

To investigate the role of configurational entropy in the mechanism of sodium removal and to better understand the reasons for the difference in cycling stability between HE and LE materials, ex situ XRD tests were performed on HE‐HCF and FeMn‐HCF. As can be seen from the Figure 5c, the HE‐HCF sample exhibit the classical solid solution type behavior of high‐entropy materials, with only minor peak shifts during GCD test.^[^ ^22^, ^44^ ^]^ And during the cycling process of charging, the peak positions show a smaller shift toward higher 2‐Theta values due to the contraction of the crystal cells when embedded in Na^+^, and then they return to their initial positions during the discharging cycle. Overall, no significant shift occurs, highlighting the overall cycling reversibility, compared to conventional HCFs, no phase transition occurs during the entire measurement process.^[^ ^45^ ^]^ Figure 5d depicts the 200 and 220 reflections, and no relatively large changes occurred. In contrast, the difference is more pronounced for FeMn‐HCF (Figure 5e). The structure is significantly destabilized compared to HE‐HCF, as shown in the Figure 5f, the reflections of FeMn‐HCF at both 200 and 220 are significantly less stable than those of HE‐HCF, and it is evident that a partial phase change has occurred during the measurement process, which may be responsible for the capacity decay of FeMn‐HCF.

**Figure 6:**
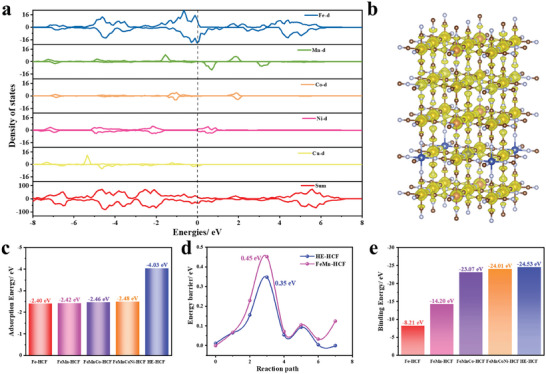
a) The TDOS and PDOS of metals in HE‐HCF. b) The partial charge density isosurface of HE‐HCF at 0.03 e Bohr^−3^. c) Calculation of the adsorption energy of Na^+^ on the sample surface. d) Migration barrier energies for paths in different HCFs. e) Calculation of the formation energy of different HCFs.

Theoretical calculations were next used to investigate the reaction mechanism of the HE‐HCF. The calculated XRD results matched the experimental results in Figure S42 (Supporting Information), thus confirming the confidence of the DFT calculations. In addition, the reactivity and redox potential of each transition metal can be simply obtained based on the different d band positions. **Figures** **6**a and S43 (Supporting Information) display the total density of states (TDOS) and projected density of states (PDOS) of HE‐HCF, the ME‐HCFs, and LE‐HCFs. The PDOS of HE‐HCF is mainly contributed by metal d orbitals with high‐entropy effect at the M_1_ site. Fe element has a large density of states occupying the vicinity of the Fermi energy level compared to the other transition metals and has a strong charge transfer capability, thus it is the first redox site during sodium insertion. The Ni and Cu elements exhibit relatively smaller DOS peaks than Fe and thus are subsequent reaction sites after Fe. The sodiation of Mn follows those of the Ni and Cu. The DOS peak of Co is relatively furthest from the Fermi energy level. These results can also be verified by partial charge analysis (from 0 to 4 eV above the Fermi energy level) as shown in Figure 6b. The Co shows a small amount of charge distribution in the region.^[^ ^22a^ ^]^ Moreover, We developed and optimized adsorption structural models and found that the structures of HE, ME and LE‐HCFs have different degrees of adsorption capacity for sodium ions. The computational results are illustrated in Figure 6c, high‐entropy HCF exhibit the largest negative Na^+^ adsorption energy (−4.03 eV) compared with ME‐HCFs (−2.48 and −2.46 eV) and LE‐HCFs (−2.42 and −2.40 eV), indicating that the framework structure of HE‐HCF has the strongest Na^+^ trapping ability. Figure 6d exhibits the migration energy barriers for HE‐HCF and FeMn‐HCF as a mechanistic demonstration. As a result, the Na^+^ migration energy barriers of HE‐HCF and FeMn‐HCF were calculated to be 0.35 and 0.45 eV, respectively. Therefore, the lower migration barriers in HE‐HCF are more favorable for the diffusion of sodium ions compared to FeMn‐HCF. This is because the highly crystallized structure can provide smoother solid‐state ion diffusion pathways, while conformational modification of the highly reversible zero‐strain Na^+^ storage mechanism is essential for suppressing the Na^+^ migration energy barrier.^[^ ^22^, ^46^ ^]^ This allows rapid Na^+^ transfer through the channels of the framework with less hindrance, elucidating the origin of rapid Na^+^ diffusion in HE‐HCF. Finally, the formation energies of HE‐HCF, ME‐HCFs and LE‐HCFs materials are calculated separately and understand the effect of configurational entropy on stability. For the desodium state before sodiumation, it is found that the formation energy of HE‐HCF is always the largest as shown in Figure 6e, suggesting that the entropy gain gives HE‐HCF the most stable structure in these samples.^[^ ^13^ ^]^

## 3 Conclusion

By introducing the concept of high‐entropy into the PBAs and obtaining entropic electrodes with different configurations of the entropy engineering strategy. Benefiting from the entropy‐dominated phase stabilization, HE‐HCF with high configurational entropy shows excellent desalination capacity and cycling stability when used as a CDI electrode based on its unique high‐entropy effect. It is proved that increasing the configurational entropy in the system can effectively inhibit phase transition in the reaction process. At the same time, the configuration modifications in the HE‐HCF system can significantly suppress the Na^+^ migration energy barrier, provide a highly reversible Na^+^ insert/extract mechanism and accelerates reaction kinetics, which results in a competitive desalination capacity and cycling lifespan. This entropy engineering including high entropy strategy provides a new way to solve the problem that it is difficult to achieve efficient coexistence of capacity and cycling stability of PBAs for high performance CDI applications.

## Conflict of Interest

The authors declare no conflict of interest.

## Supporting information

Supporting Information

## Data Availability

The data that support the findings of this study are available in the supplementary material of this article.
