# Supplementary material for: Entropy Engineering Constrain Phase Transitions Enable Ultralong‐life Prussian Blue Analogs Cathodes
Source: Adv Sci (Weinh). 2024 Apr 26;11(28):2402340. doi: 10.1002/advs.202402340 (PMC11267327; doi:10.1002/advs.202402340)
Supplement: Supplementary file 1 — Supporting Information [file ADVS-11-2402340-s001.pdf]

ADVANCED SCIENCE
Open Access

Supporting Information

for *Adv. Sci.*, DOI 10.1002/advs.202402340

Entropy Engineering Constrain Phase Transitions Enable Ultralong-life Prussian Blue Analogs Cathodes

Yuhao Lei, Shiyong Wang, Lin Zhao, Changping Li, Gang Wang* and Jieshan Qiu*

Supporting Information

Entropy Engineering Constrain Phase Transitions Enable Ultralong-life Prussian Blue Analogs Cathodes

Yuhao Lei [a1], Shiyong Wang [a1*], Lin Zhao [a, b]; Changping Li [a]; Gang Wang [a*]; Jieshan Qiu [b]

[a] School of Environment and Civil Engineering, Research Center for Eco-environmental Engineering, Dongguan University of Technology, Dongguan 523106, Guangdong, PR China

[b] College of Chemical Engineering, Beijing University of Chemical Technology, Beijing 100029, PR China

Methods

Materials Synthesis. High-entropy Prussian blue analoge (HE-HCF) was synthesized by co-precipitation method. 2 mmol $Fe(NO_3)_3·9H_2O$, 2 mmol $Mn(NO_3)_2·4H_2O$, 2 mmol $Co(NO_3)_2·6H_2O$, 2 mmol $Ni(NO_3)_2·6H_2O$, $Cu(NO_3)_2·3H_2O$ and 10 mmol sodium citrate were dissolved in 100 ml deionized (DI) water to form solution A, and stirred thoroughly, and 15 mmol $Na_4Fe(CN)_6$ was dissolved in deionized (DI) water to form solution B, and stirred until well dispersed. The B solution was slowly dripped into the A solution using a dispensing funnel under continuous stirring, and the mixed solution was then aged at room temperature for 24 h. The products were collected by centrifugation and washed with deionized water and ethanol for several times, respectively, and then the final product was dried in a vacuum oven at 120 °C for 12 h.

* Corresponding Authors
E-mail: wsyongcqu@126.com (SY Wang)
wghy1979@163.com (G Wang)
[1] Lei and Wang equal contribution to this work

The medium-entropy Prussian blue analogs (FeMnCoNi-HCF、FeMnCo-HCF) and the low-entropy Prussian blue analogs (FeMn-HCF、Fe-HCF) were prepared analogously by using the respective metal precursors.

Characterization. The crystal structure of the samples were investigated via powder X-ray diffraction (XRD) using either a Bruker D8 Advance (Cu-Kα1 radiation, λ = 1.54056 Å). The diffraction pattern was first indexed and then refined by the Rietveld method with GSAS software. The morphologys were characterized by scanning electron microscopy (SEM, VeriosG4UC) and transmission electron microscopy (TEM, TALOS F200S G2). The elemental energy dispersive spectroscopy (EDS) mapping images were obtained through STEM. The Fourier transform infrared (FTIR) spectras were recorded with a FTIR spectrometer (FRONTIER). X-ray photoelectron spectroscopy (XPS) with an Al Kα source was applied to collect the surface information through an ESCALAB XI spectrometer. The X-ray absorption spectra (XAS) including X-ray absorption near-edge structure (XANES) and extended X-ray absorption fine stucture (EXAFS) of the samples at Fe, Mn, Co, Ni, Cu K-edge were colleted at the Singapore Synchrotron Light Source (SSLS) center, where a pair of chanel-cut Si (111) crystals was used in the monochromator.

Calculation of configurational entropy.

For a random solid solution, the configurational entropy per mole can be expressed as:[1]

$$\Delta S_{\text{conf}} = -R\sum_{i=1}^{n} x_i \qquad \textbf{(Equation S1)}$$

where R is the gas constant, 8.314 J/K mol, and n is the number of elements, and x_i represents the molar fraction of the ith component.[2]

$$\Delta S_{\text{conf}} = -R\left(\frac{1}{n}\ln\frac{1}{n}+\frac{1}{n}\ln\frac{1}{n}+\cdots+\frac{1}{n}\ln\frac{1}{n}\right) = -R\ln\frac{1}{n} = R\ln n \qquad \textbf{(Equation S2)}$$

Electrochemical Measurements. Cyclic voltammetry (CV) and electrochemical impedance spectroscopy (EIS) were conducted by using a CHI 660E electrochemical workstation in 1 M NaCl solution. A three-electrode system consisting of a platinum foil as the counter electrode, an Ag/AgCl electrode (saturated KCl) as the reference electrode, and the sample was used as the working electrode. To prepare the working electrodes, a homogeneous slurry of PBAs, polytetrafluoroethene (PTFE), and carbon black in ethanol with a mass ratio of 8:1:1 was coated on graphite paper and dried at 80 ℃ overnight. The obtained electrode had a coating surface area of 4 cm^2 with a mass loading of around 3-4 mg cm^2. The specific capacitance (C, F g-1) was calculated from the CV curves as:

$$C = \frac{\int I dt}{2\times m\times v\times \Delta v} \qquad \textbf{(Equation S3)}$$

Where C is the specific capacitance (F g^{-1}), I is the response current density (A), v is the potential scanning rate (V s^{-1}), Δv is the voltage change (V), and m is the active material mass (g).

The current response of the cyclic voltammetry curve at different voltage value and sweep speed follow the following equation:

$$i = av^b \qquad \textbf{(Equation S4)}$$

Where i is the current response value (mA), v is the sweep speed (mV s^{-1}), and a and b are suitable values. The b value is between 0.5 and 1.

The capacity contribution can be quantified according to the following equation:

$$i = k_1 v + k_2 v^{1/2} \quad \textbf{(Equation S5)}$$

where i and v denote the total current (A) and the potential scanning rate (V s^{-1}), plotting $v^{1/2}$ against $i/v^{1/2}$, the slope of the resulting line is k_1 and the intercept is k_2.

Measurements of CDI performance. To prepare the CDI electrodes,80 wt% active materials (AC, PBAs), 10 wt% carbon black, 8 wt% polyvinyl butyral (PVB), and 2 wt%polyvinylpyrrolidone (PVP) were mixed together to obtain ahomogeneous slurry. Subsequently, the resulting slurry of solids was cast on a graphite paper (4.5×6.5 cm^2) and then dried at 80 ℃ overnight. A PBAs electrode, an AC electrode, A PBAs electrode, an AC electrode, and a piece of anion exchange membrane were assembled into a CDI. During the CDI process, sodium chloride (NaCl) solution with different concentrations (100 to 1000 mg L^{-1}) was continuously pumped into the CDI unit, and returned to the feed container.The real-time monitoring of the concentration of NaCl solution was achieved by recording its conductivity with an ionic conductivity meter, where an initial concentration of 500 mg L^{-1} of NaCl solution corresponded to a conductivity of 1000 μS cm^{-1}.

The desalination capacity (Γ, mg g-1) was calculated using Equation S6:

$$\Gamma = \frac{\Phi \times \int C_0 - C_t dt}{m} \qquad \textbf{(Equation S6)}$$

where Φ is the flow rate (mL min^{-1}), C_0 and C_t are the influent and effluent NaCl concentrations (mg L^{-1}), respectively, m is the mass of active material (g).

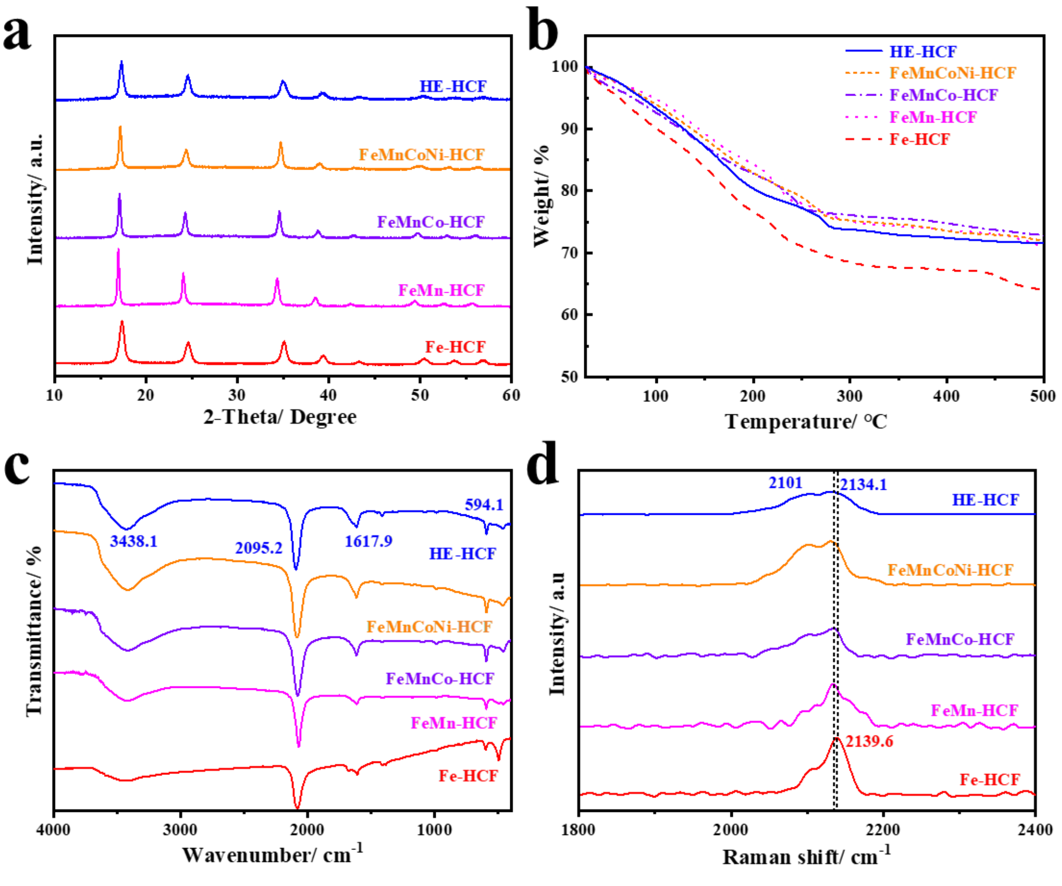

Figure S1. (a) XRD patterns. (b) TGA curves. (c) FT-IR spectra. (d) Raman spectra.

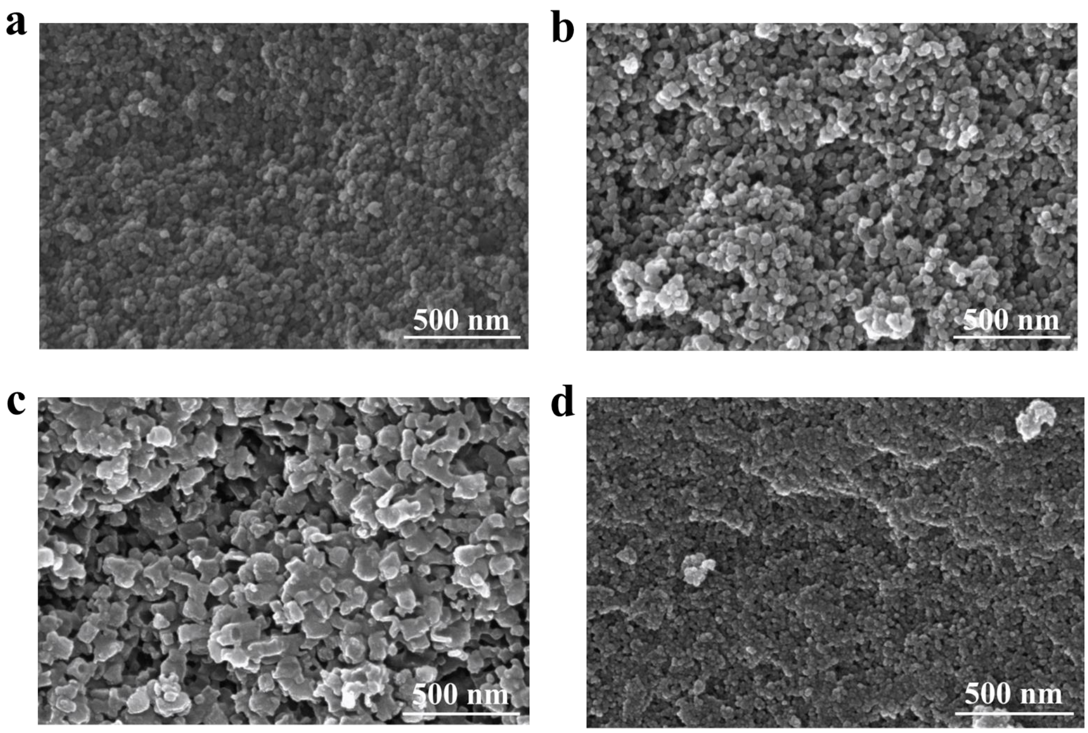

Figure S2. SEM images before cycling. (a) FeMnCoNi-HCF. (b) FeMnCo-HCF. (c) FeMn-HCF and (d) Fe-HCF.

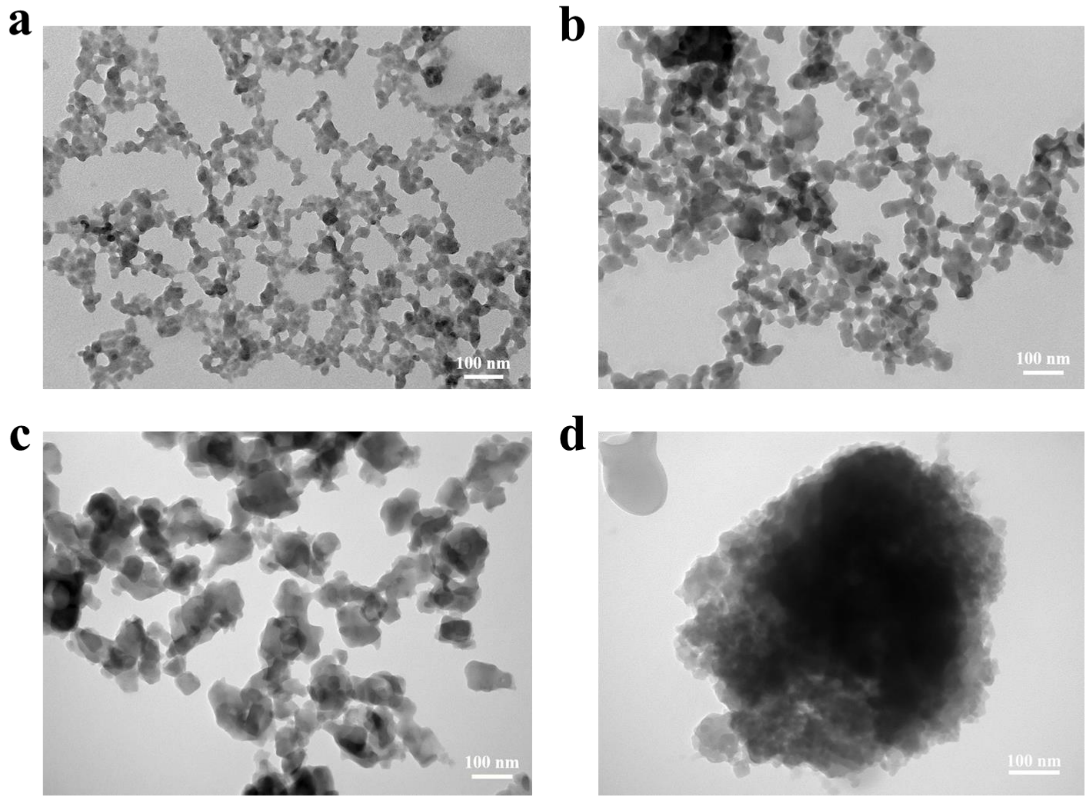

Figure S3. TEM images before cycling. (a) FeMnCoNi-HCF. (b) FeMnCo-HCF. (c) FeMn-HCF and (d) Fe-HCF.

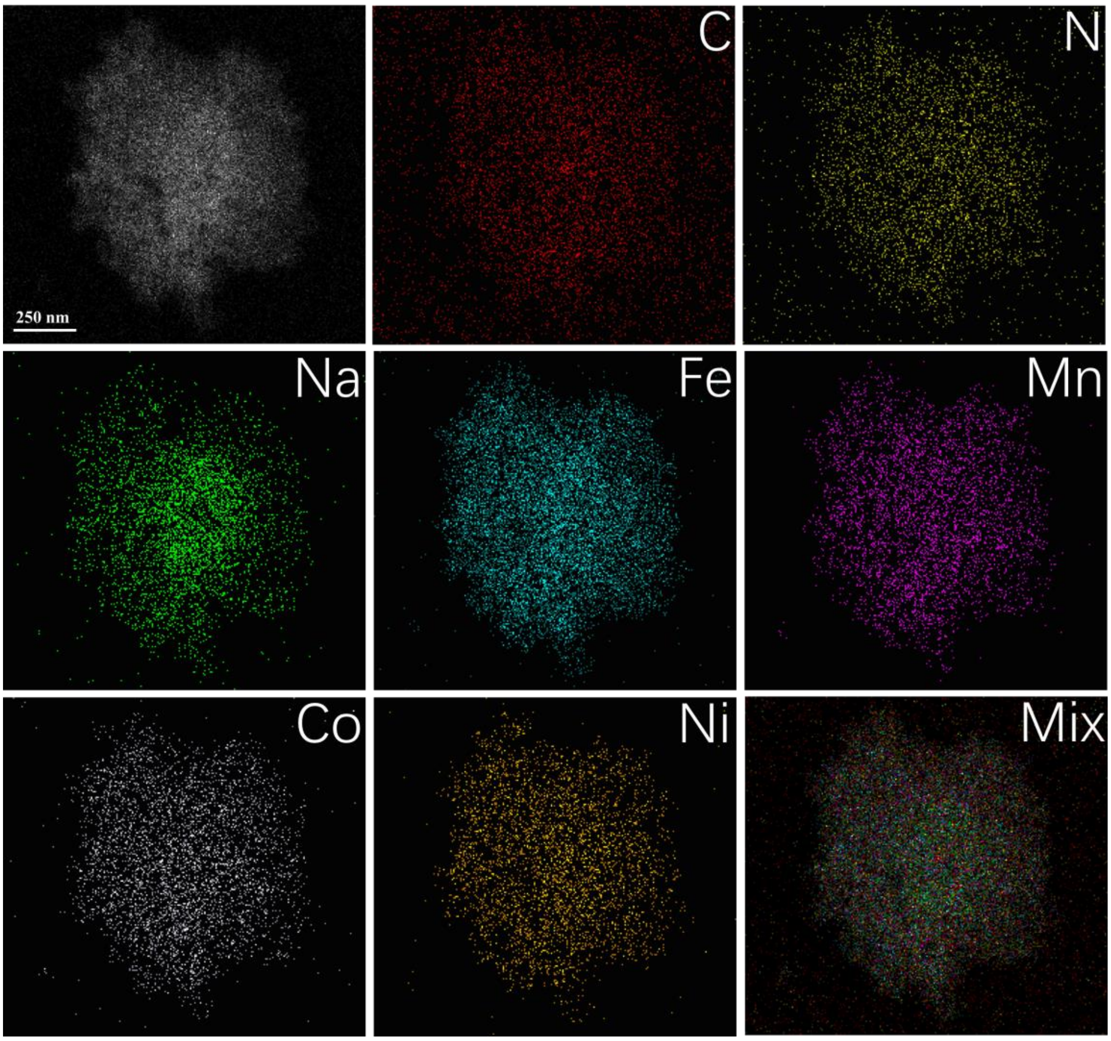

Figure S4. EDS elemental mapping images of FeMnCoNi-HCF.

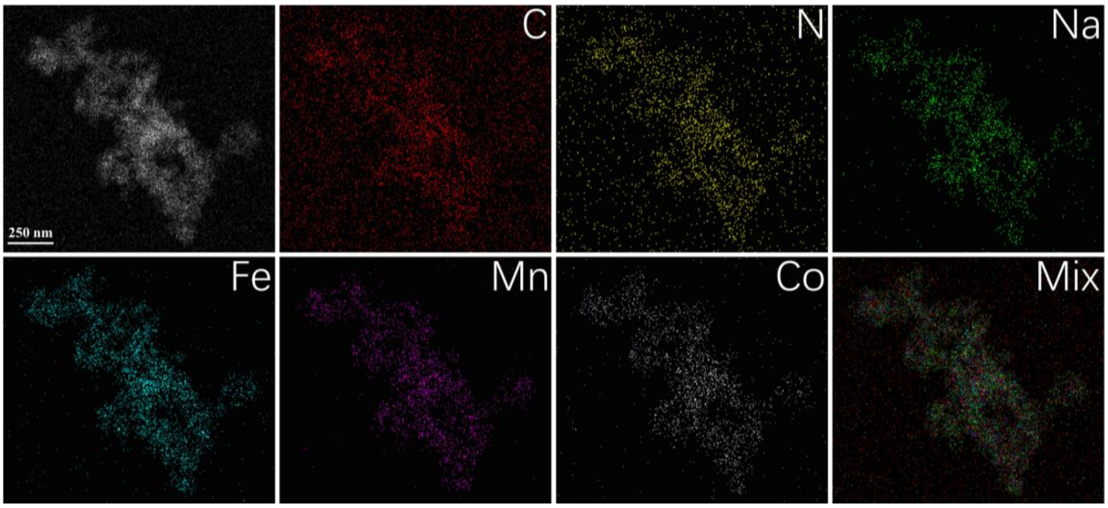

Figure S5. EDS elemental mapping images of FeMnCo-HCF.

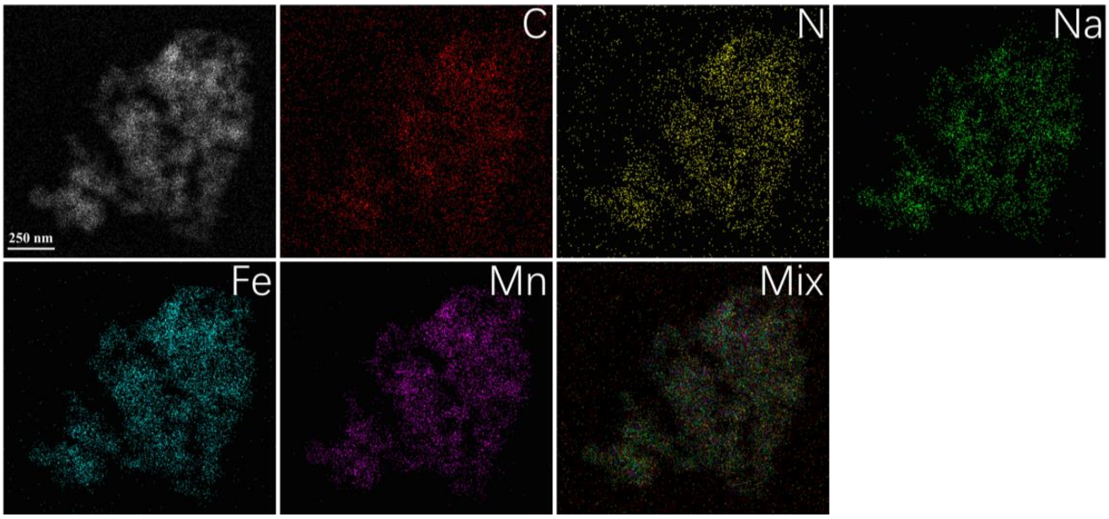

Figure S6. EDS elemental mapping images of FeMn-HCF.

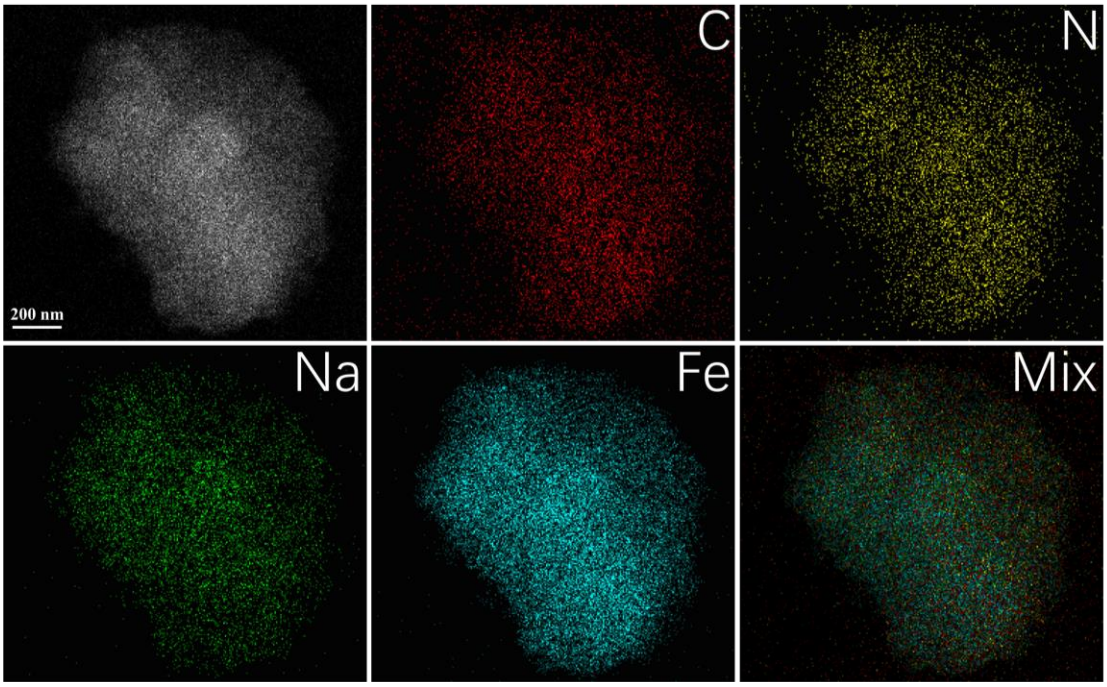

Figure S7. EDS elemental mapping images of Fe-HCF.

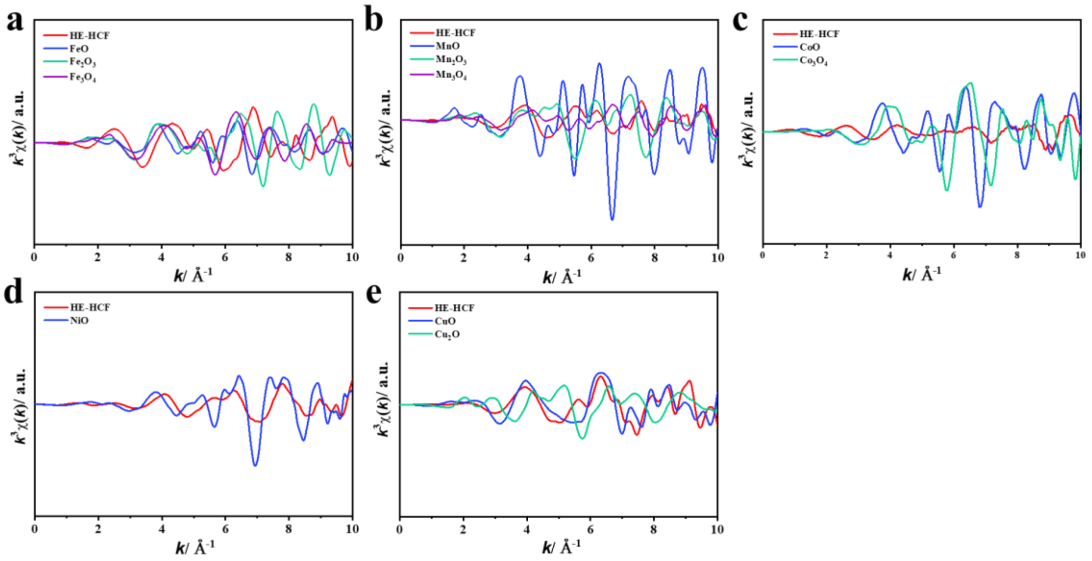

Figure S8. The K-edge EXAFS spectrum with k^3-weight in k-space for HE-HCF and corresponding standard references.

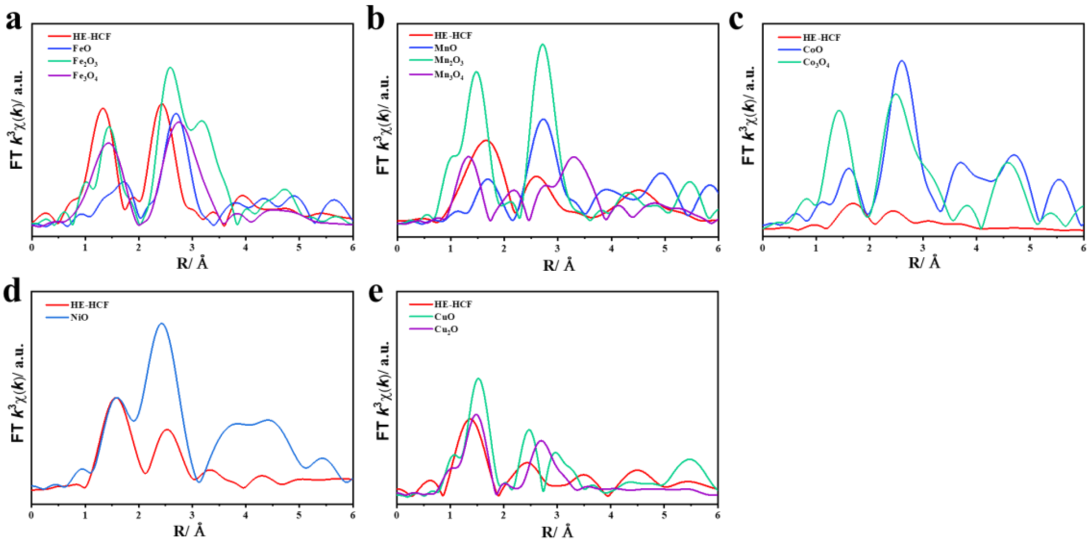

Figure S9. The Fourier transform of the EXAFS spectrum of (a) Fe K-edge, (b) Mn K-edge, (c) Co K-edge, (d) Ni K-edge, and (e) Cu K-edge of HE-HCF and corresponding standard reference samples.

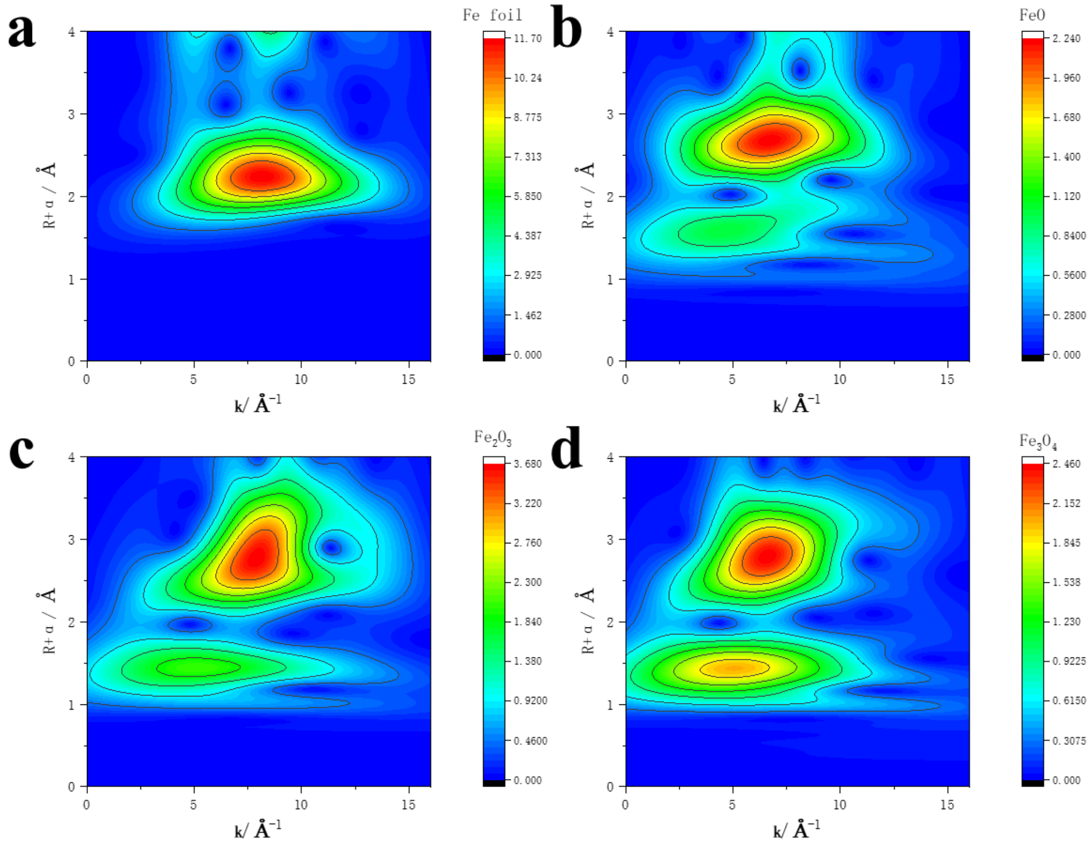

Figure S10. Wavelet transforms (WT) of (a) Fe foil. (b) FeO. (c) Fe_2O_3. (d) Fe_3O_4.

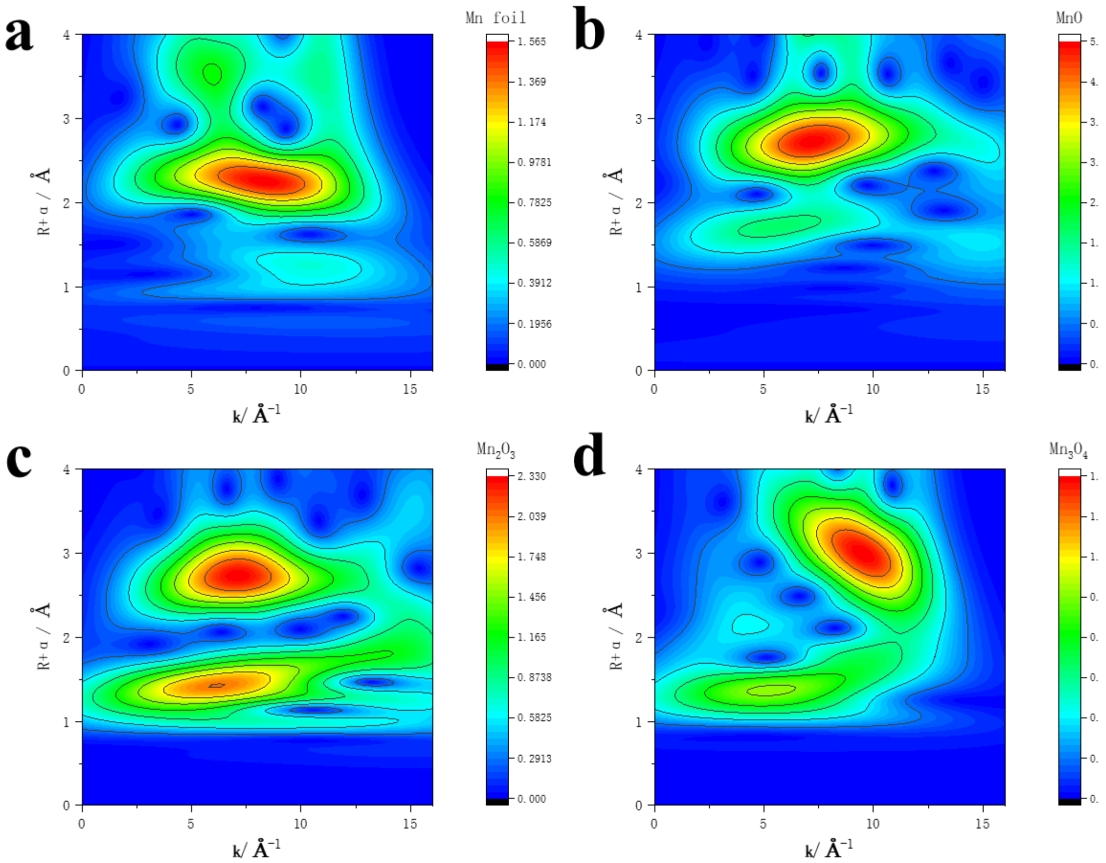

Figure S11. Wavelet transforms (WT) of (a) Mn foil. (b) MnO. (c) Mn_2O_3. (d) Mn_3O_4.

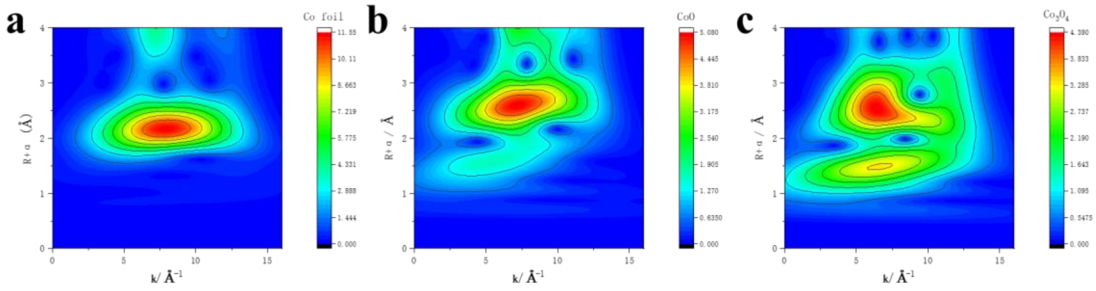

Figure S12. Wavelet transforms (WT) of (a) Co foil. (b) CoO. (c) Co_3O_4.

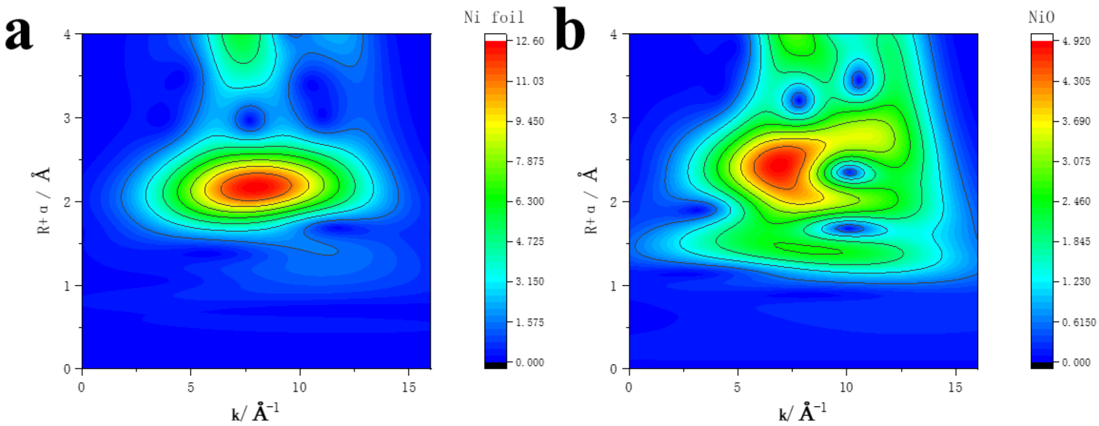

Figure S13. Wavelet transforms (WT) of (a) Ni foil. (b) NiO.

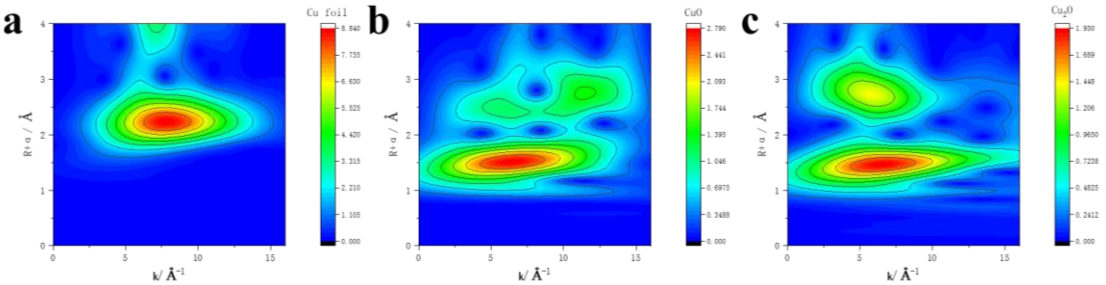

Figure S14. Wavelet transforms (WT) of (a) Cu foil. (b) CuO. (c) Cu_2O.

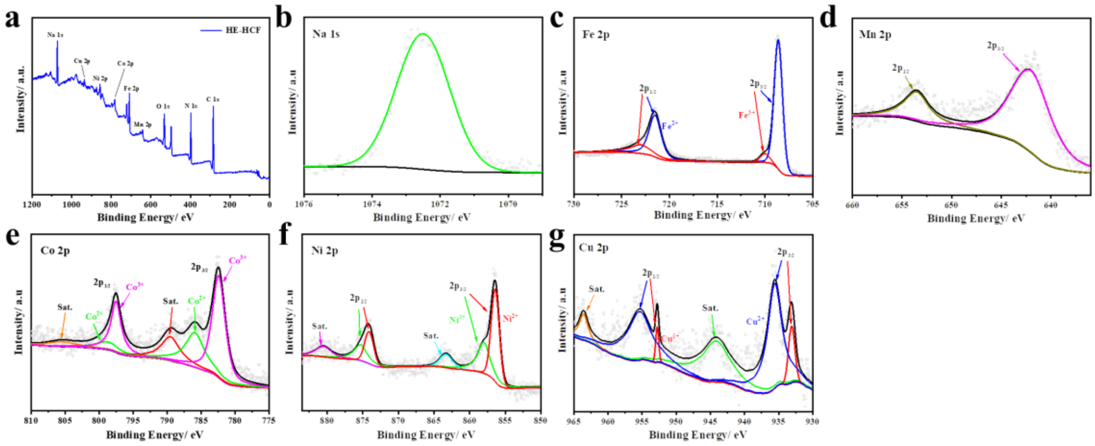

Figure S15. XPS survey spectrum of HE-HCF of (a) XPS survey spectra. (b) Na 1s. (c) Fe 2p. (d) Mn 2p. (e) Co 2p. (f) Ni 2p and (g) Cu 2p.

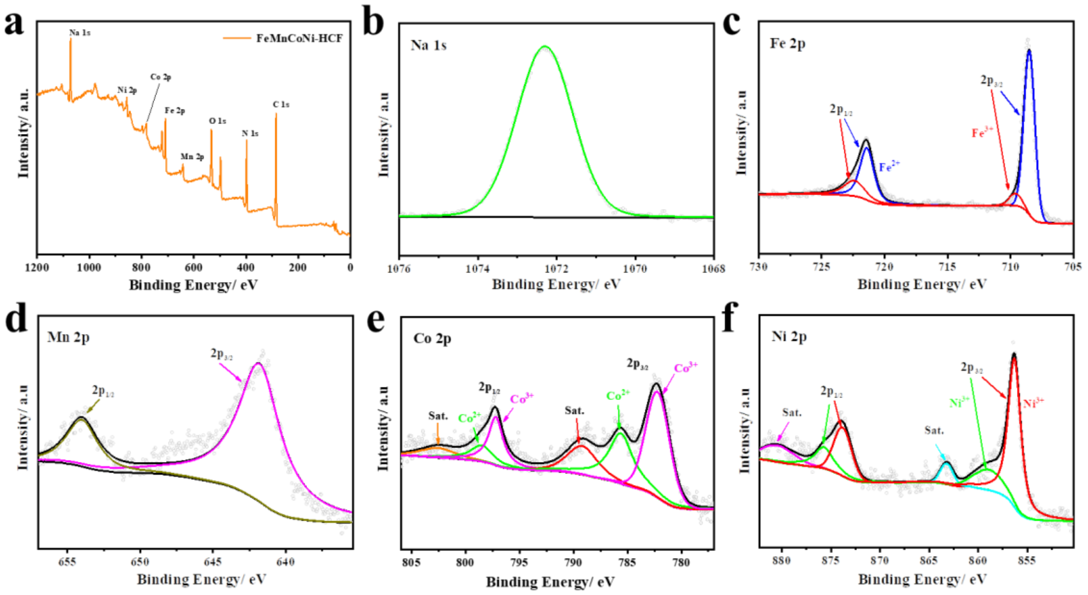

Figure S16. XPS survey spectrum of FeMnCoNi-HCF of (a) XPS survey spectra. (b) Na 1s. (c) Fe 2p. (d) Mn 2p. (e) Co 2p and (f) Ni 2p.

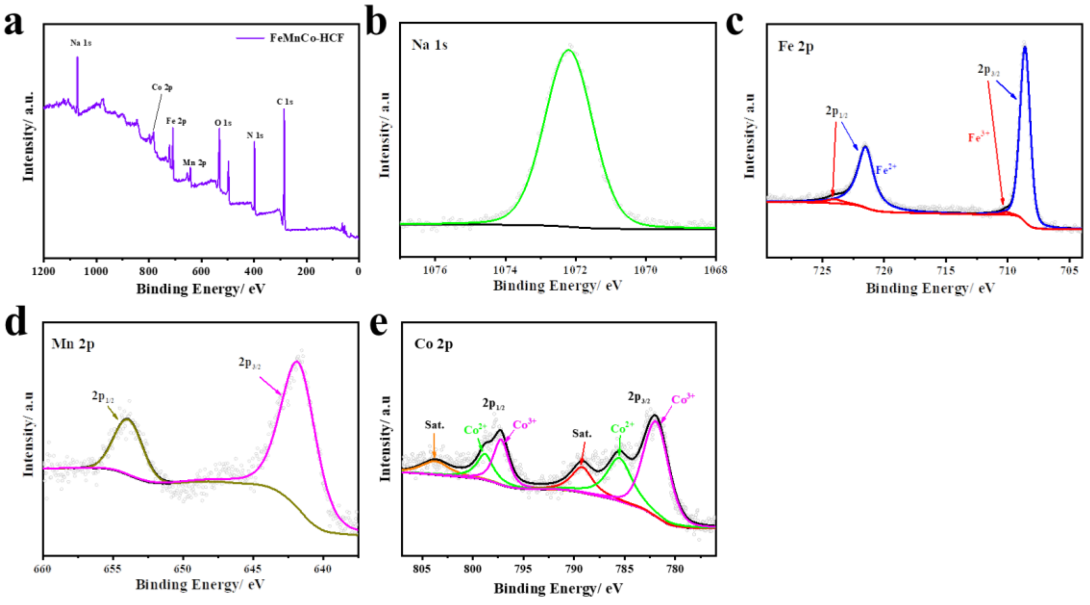

Figure S17. XPS survey spectrum of FeMnCo-HCF of (a) XPS survey spectra. (b) Na 1s. (c) Fe 2p. (d) Mn 2p and (e) Co 2p.

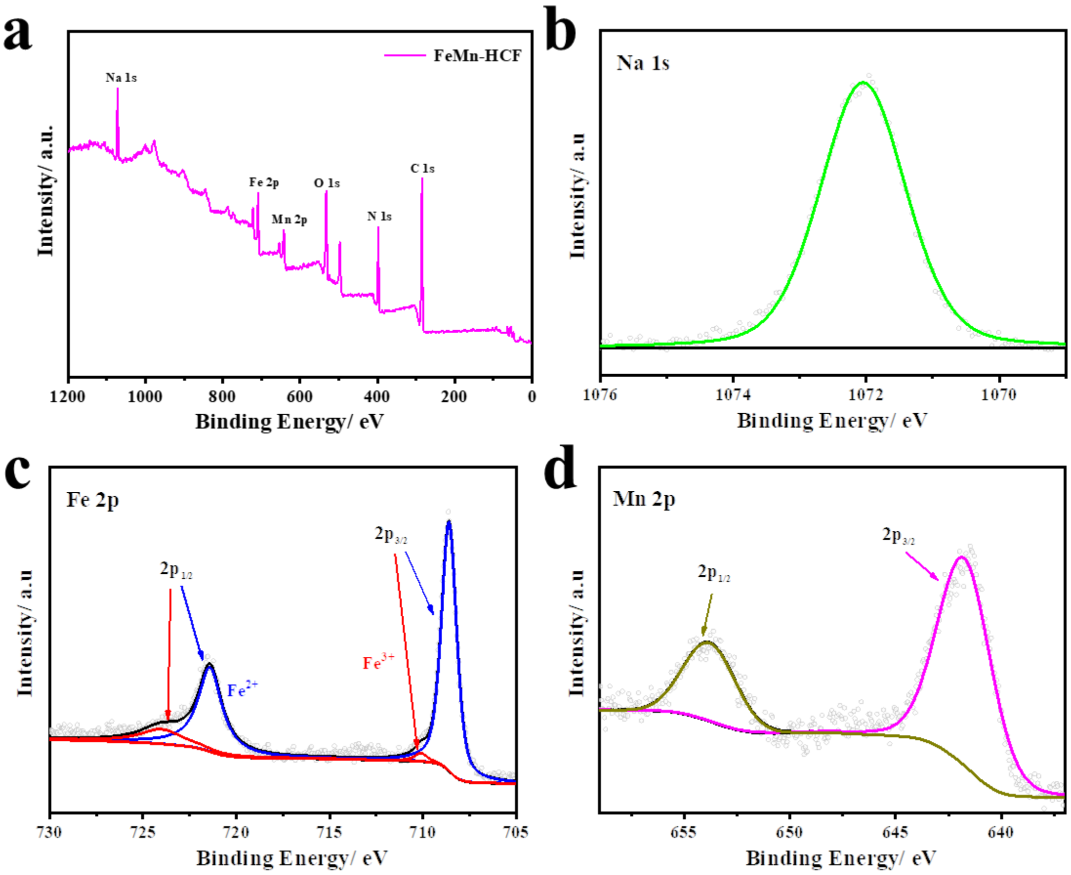

Figure S18. XPS survey spectrum of FeMn-HCF of (a) XPS survey spectra. (b) Na 1s. (c) Fe 2p and (d) Mn 2p.

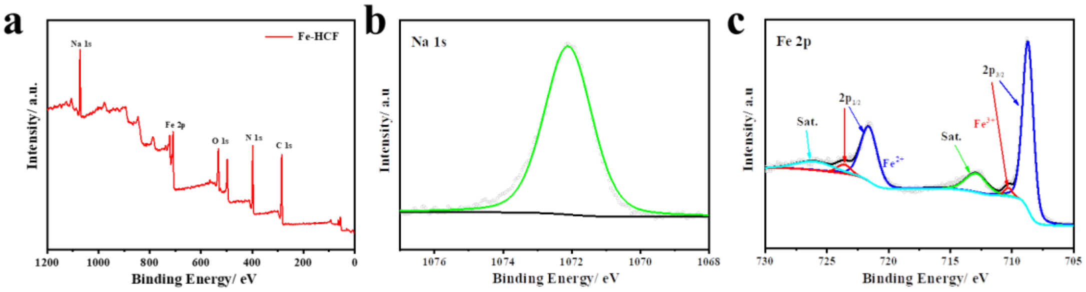

Figure S19. XPS survey spectrum of Fe-HCF of (a) XPS survey spectra. (b) Na 1s and (c) Fe 2p.

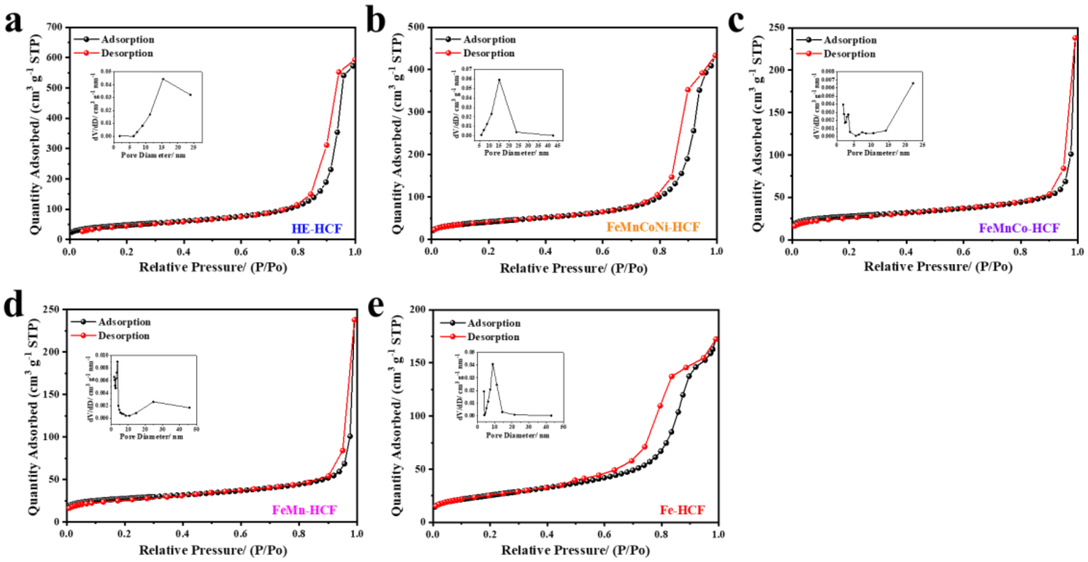

Figure S20. N_2 adsorption-desorption isotherms and pore size distributions of different HCFs.

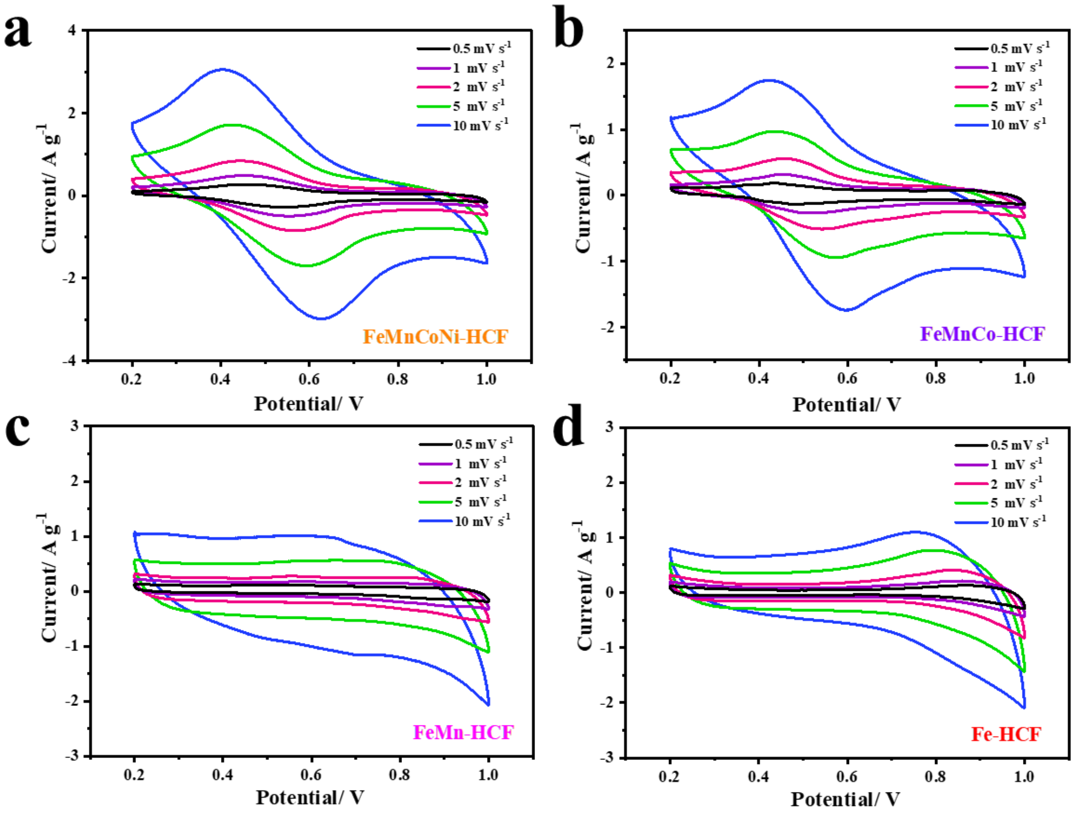

Figure S21. CV curves at different scan rates of different HCFs

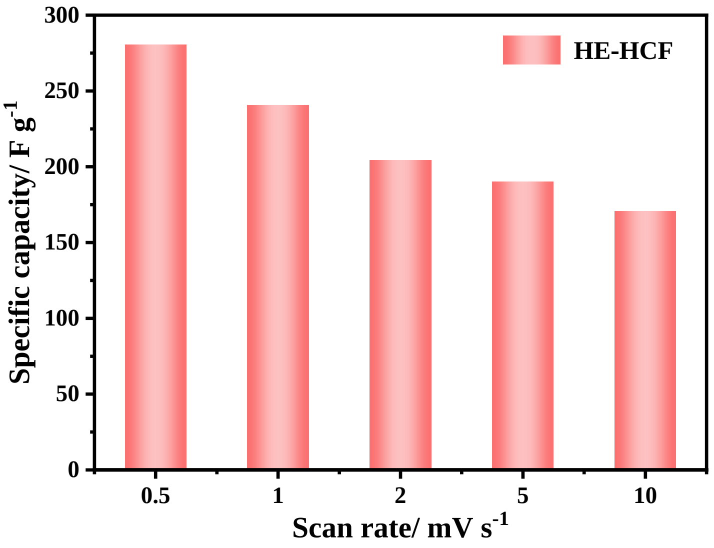

Figure S22. Specific capacitance value of HE-HCF.

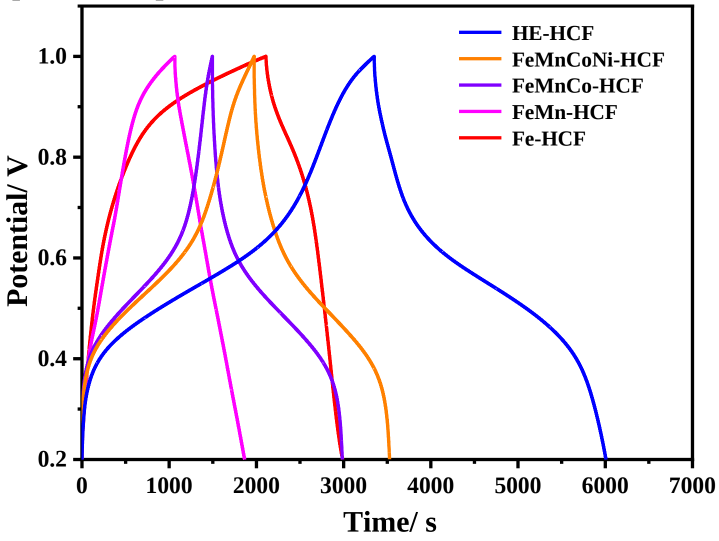

Figure S23. GCD curves at a current density of 0.1 A g^{-1}.

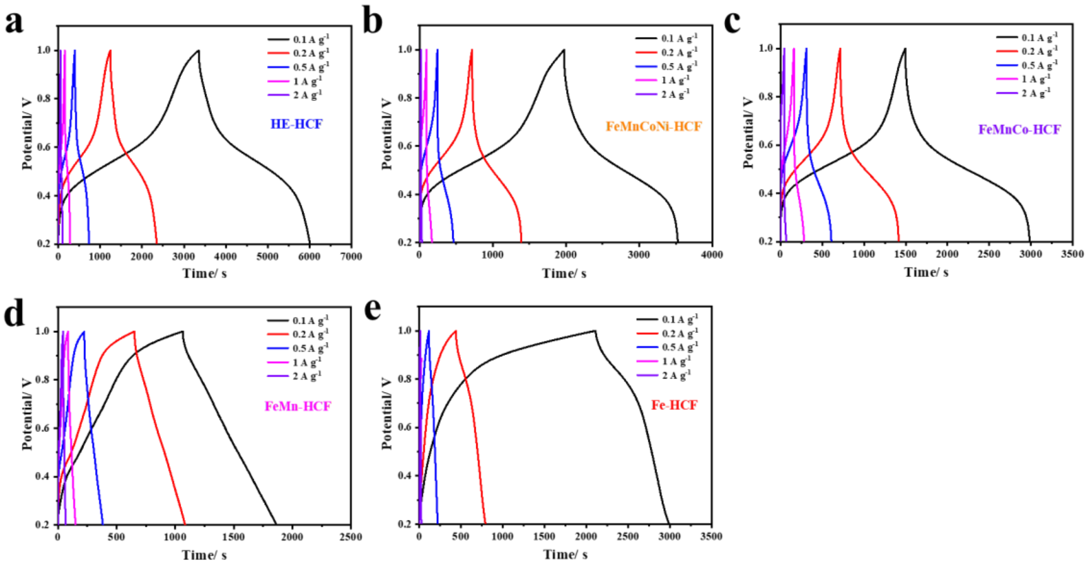

Figure S24. GCD curves at different scan rates of different HCFs.

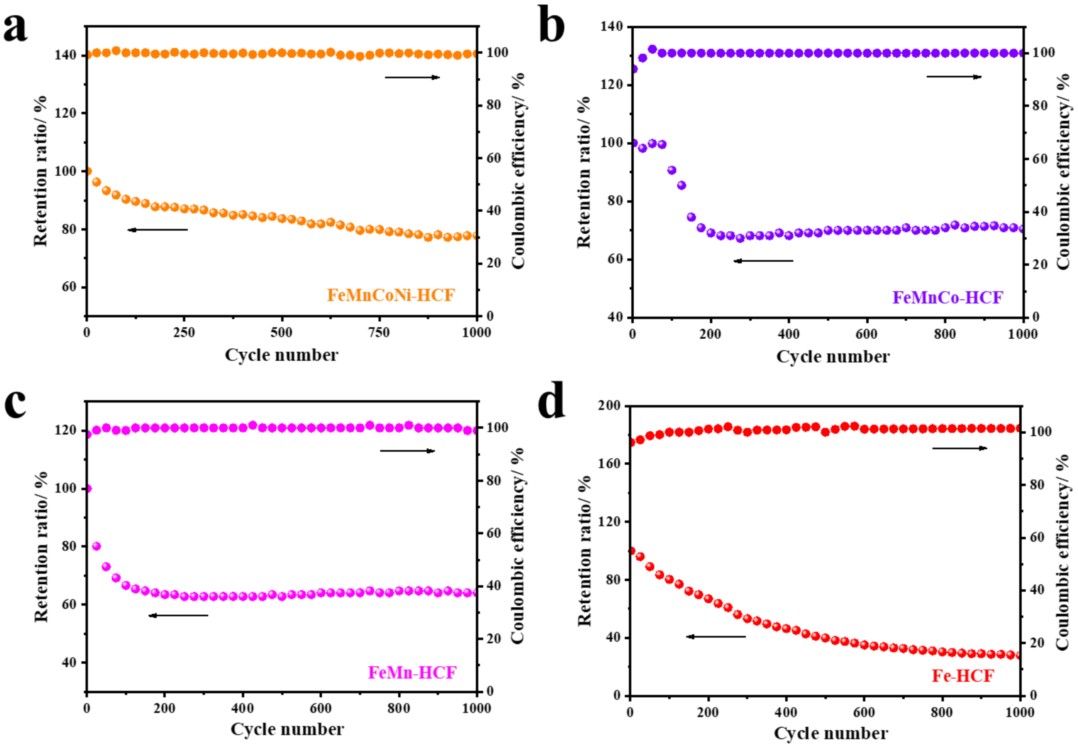

Figure S25. Cycling performance and coulombic efficiency at 2 A g^{-1} for 1000 GCD cycles of different HCFs.

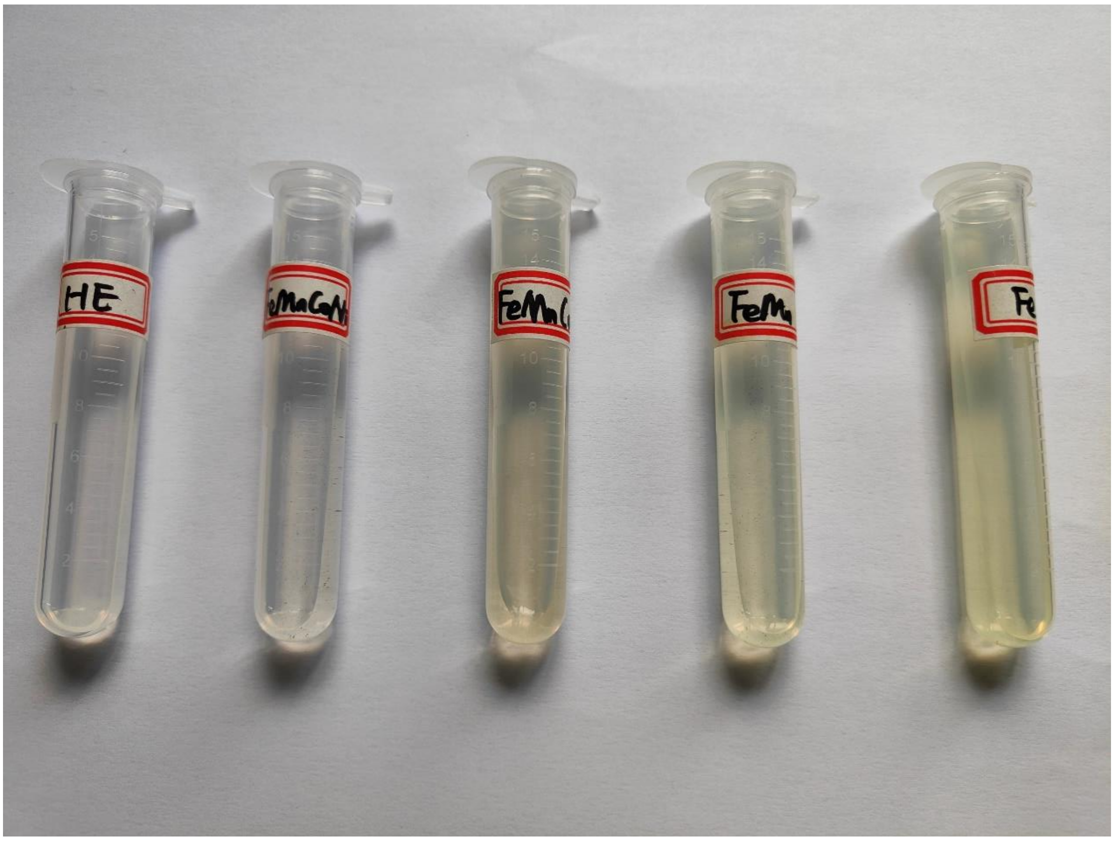

Figure S26. The color of electrolytes from different HCFs after 1000 GCD cycling.

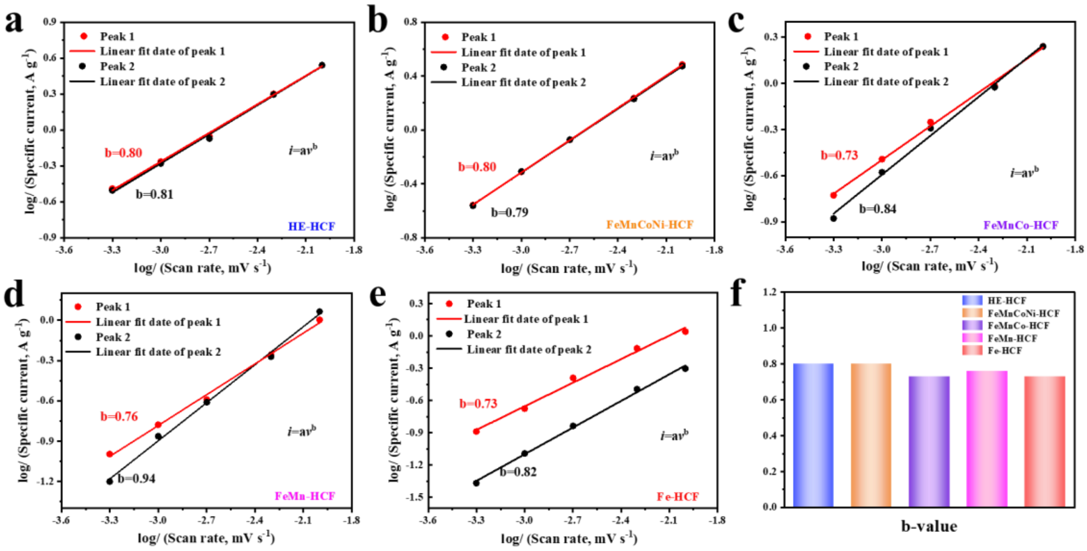

Figure S27. Linear correlation between the logarithmic peak current and sweep rate of different HCFs.

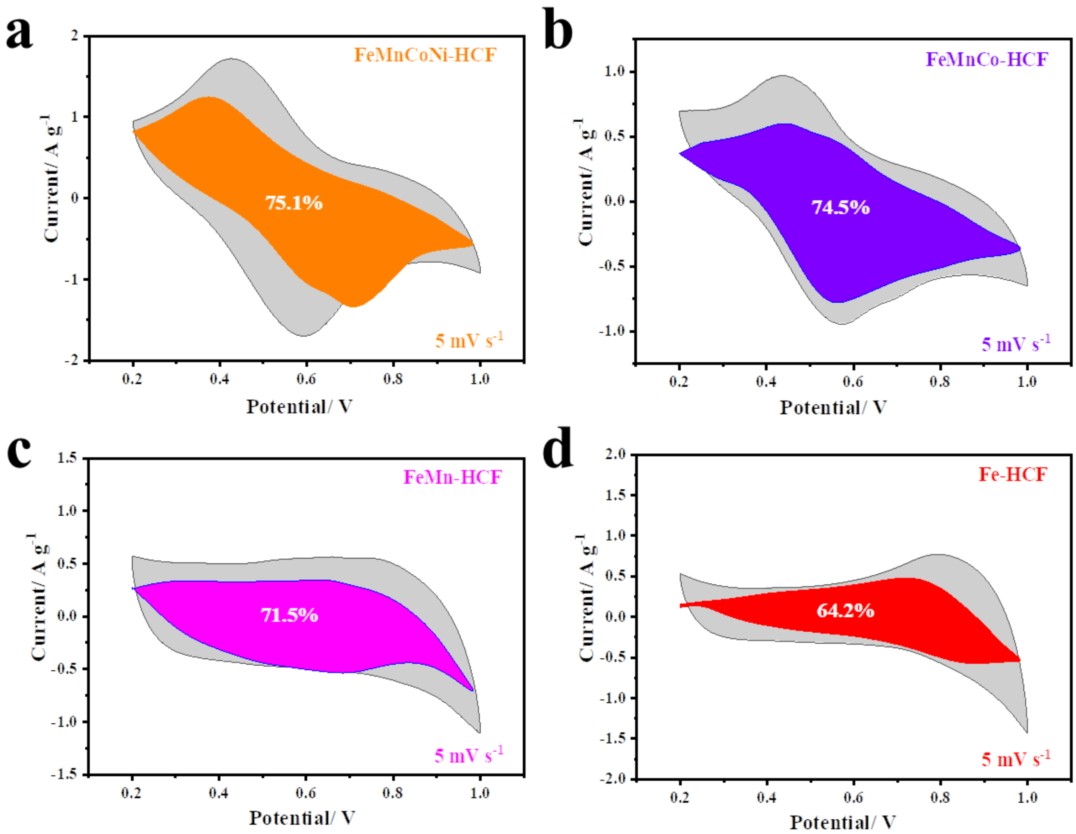

Figure S28. The contribution of capacitive-controlled (color) and diffusion-controlled process (grey) of different HCFs.

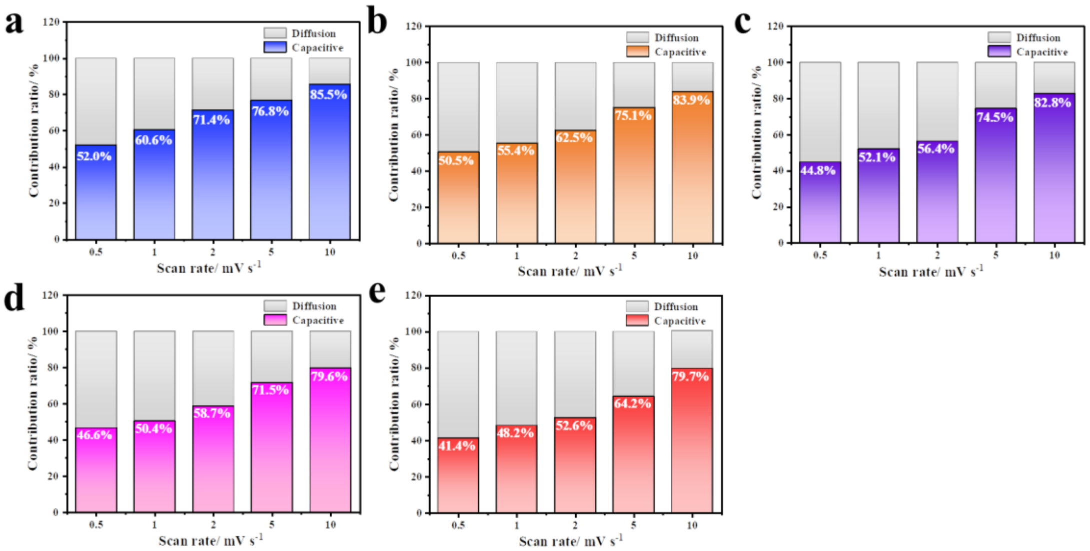

Figure S29. Normalized contribution ratios of different HCFs at different scan rates.

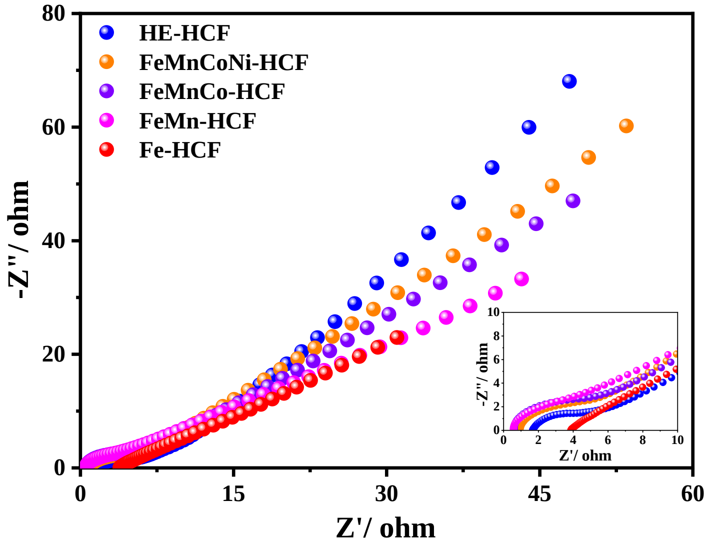

Figure S30. EIS curves of different HCFs.

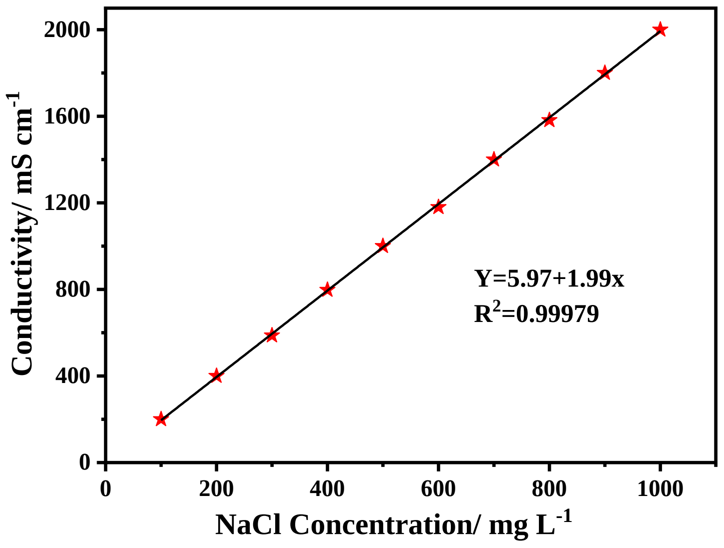

Figure S31. The relationship curve between concentration of NaCl solution and conductivity.

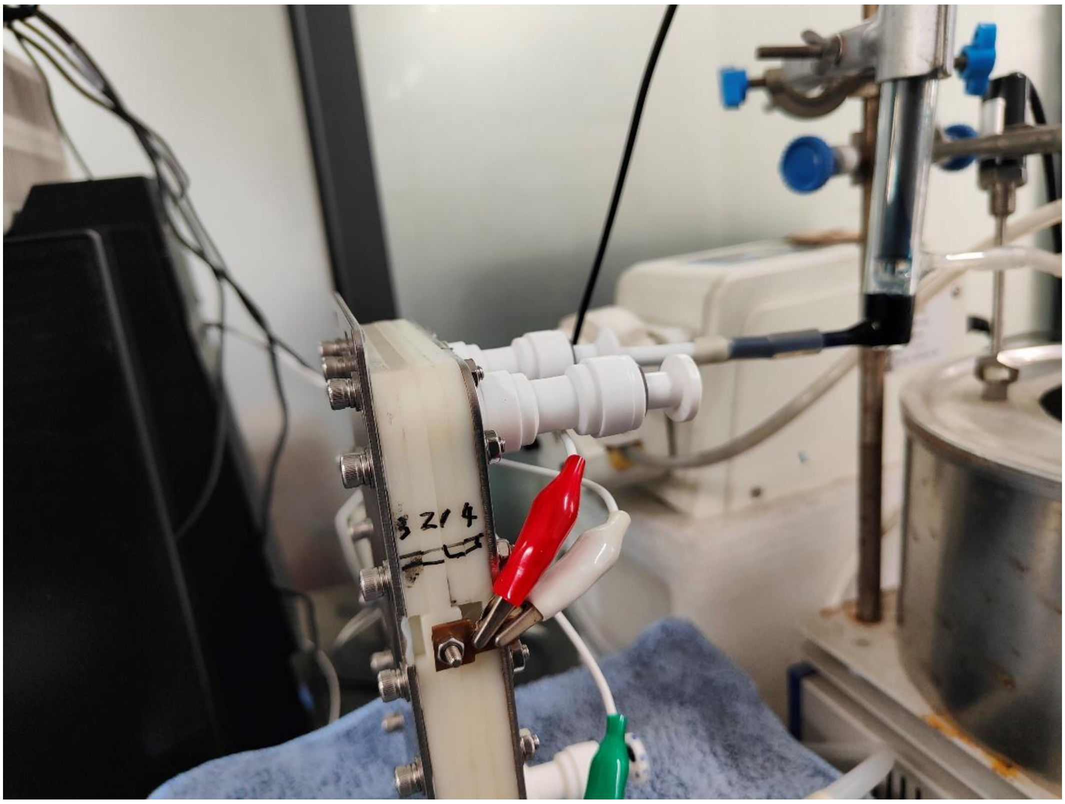

Figure S32. Fe-HCF undergoes severe discoloration when exposed to water.

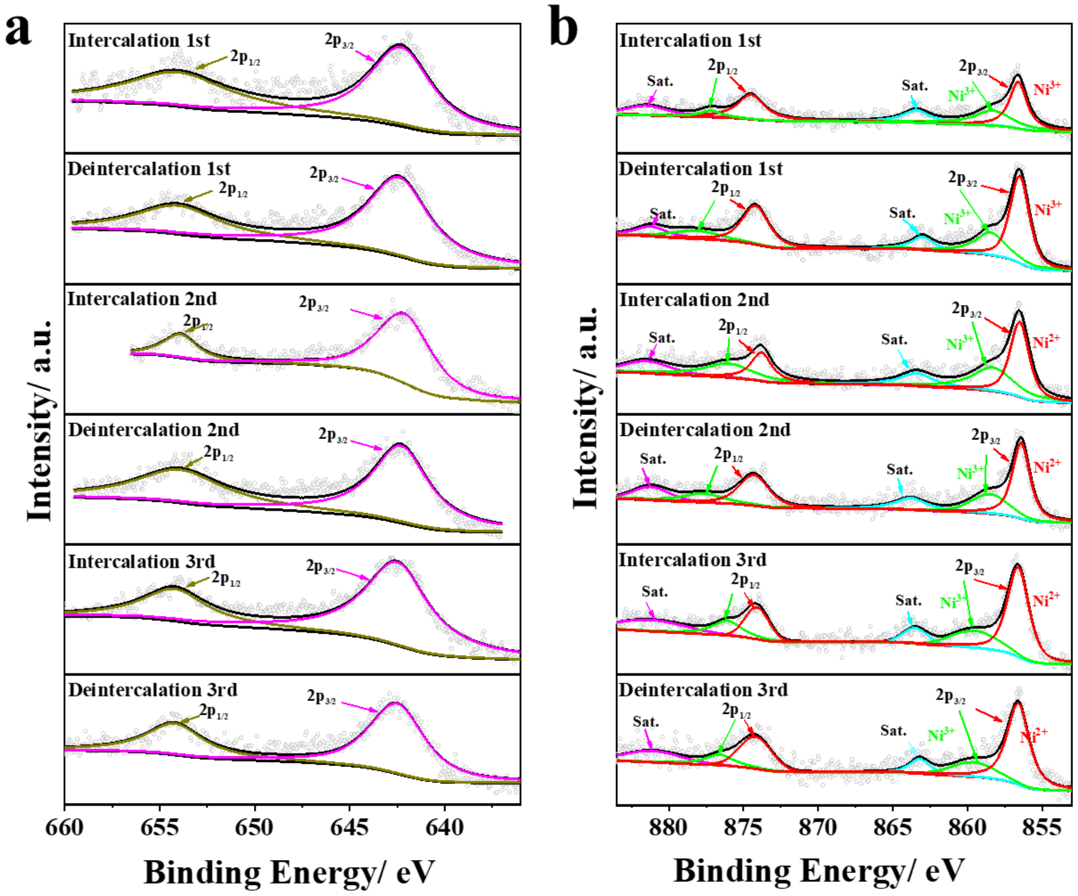

Figure S33. The XPS spectra of (a) Mn 2p and (b) Ni 2p during Na^+ intercalation and deintercalation in 1st, 2nd and 3rd cycles.

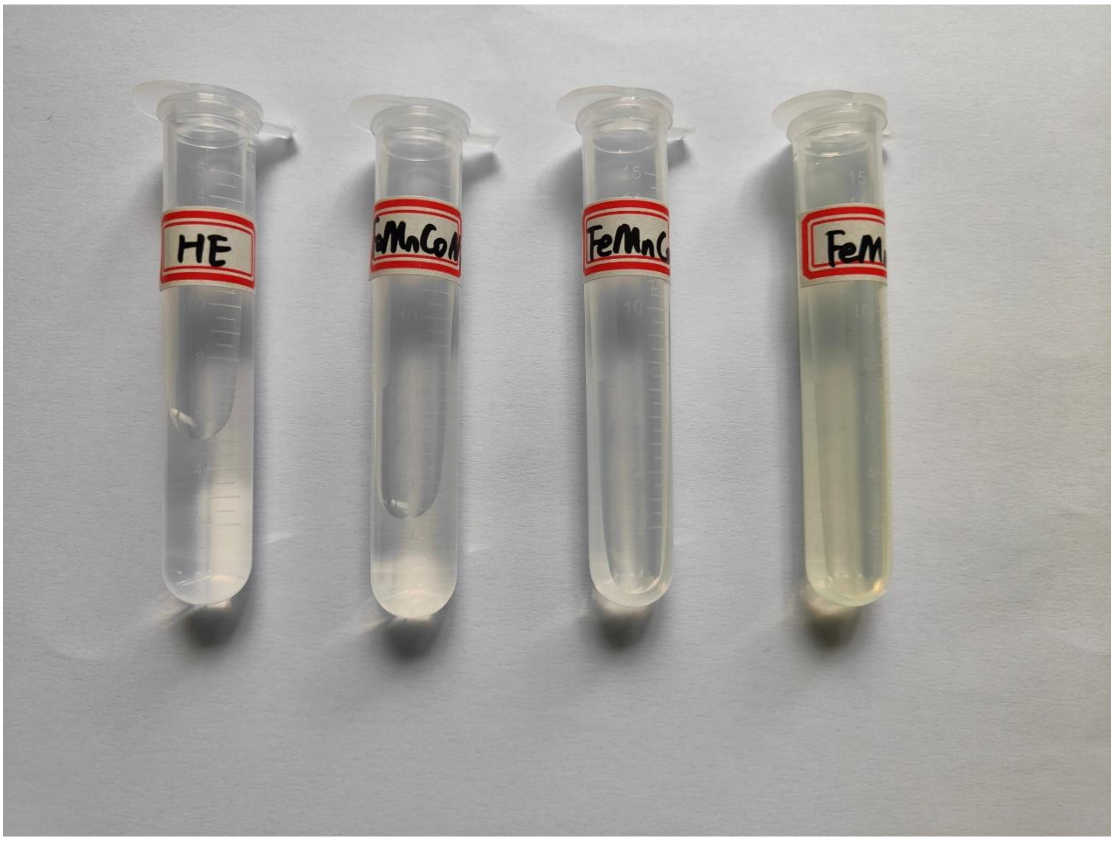

Figure S34. The color of NaCl solution from different HCFs after cycling.

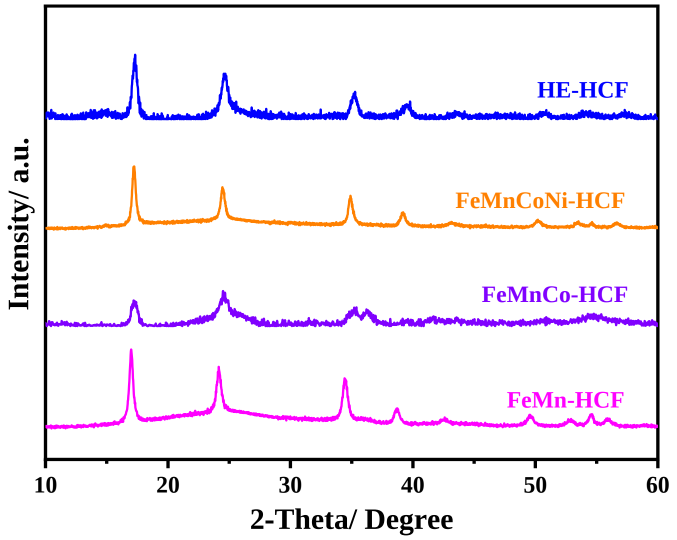

Figure S35. XRD patterns after cycling of different HCFs.

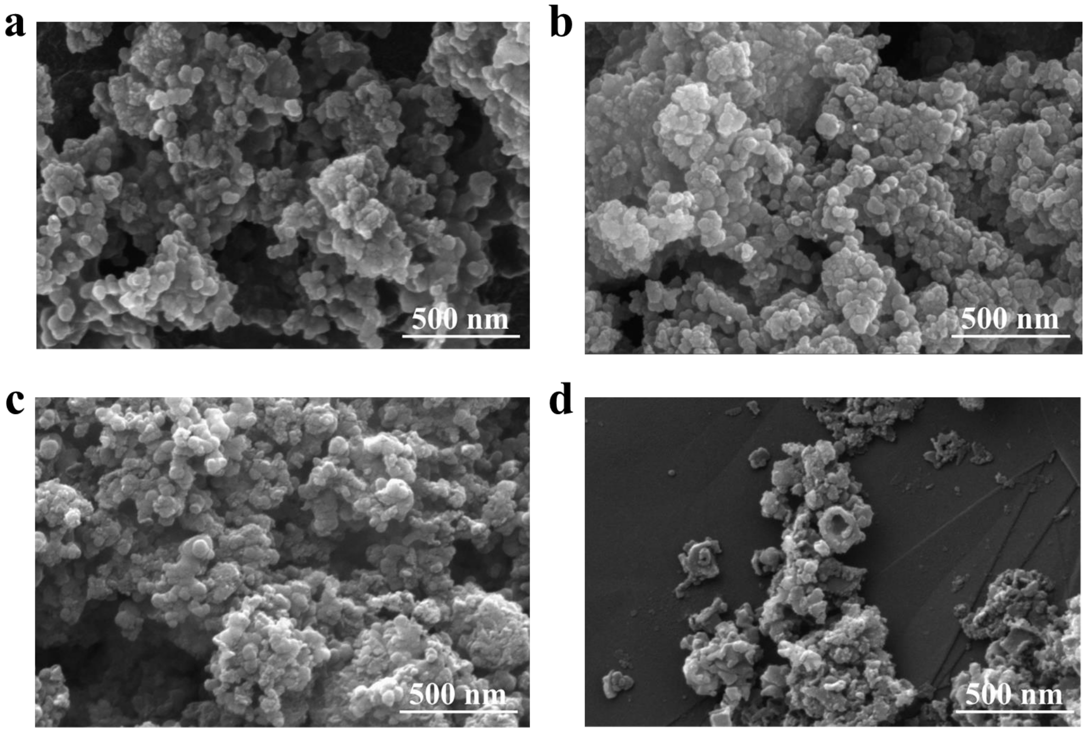

Figure S36. SEM images after cycling. (a) HE-HCF. (b) FeMnCoNi-HCF. (c) FeMnCo-HCF and (d) FeMn-HCF.

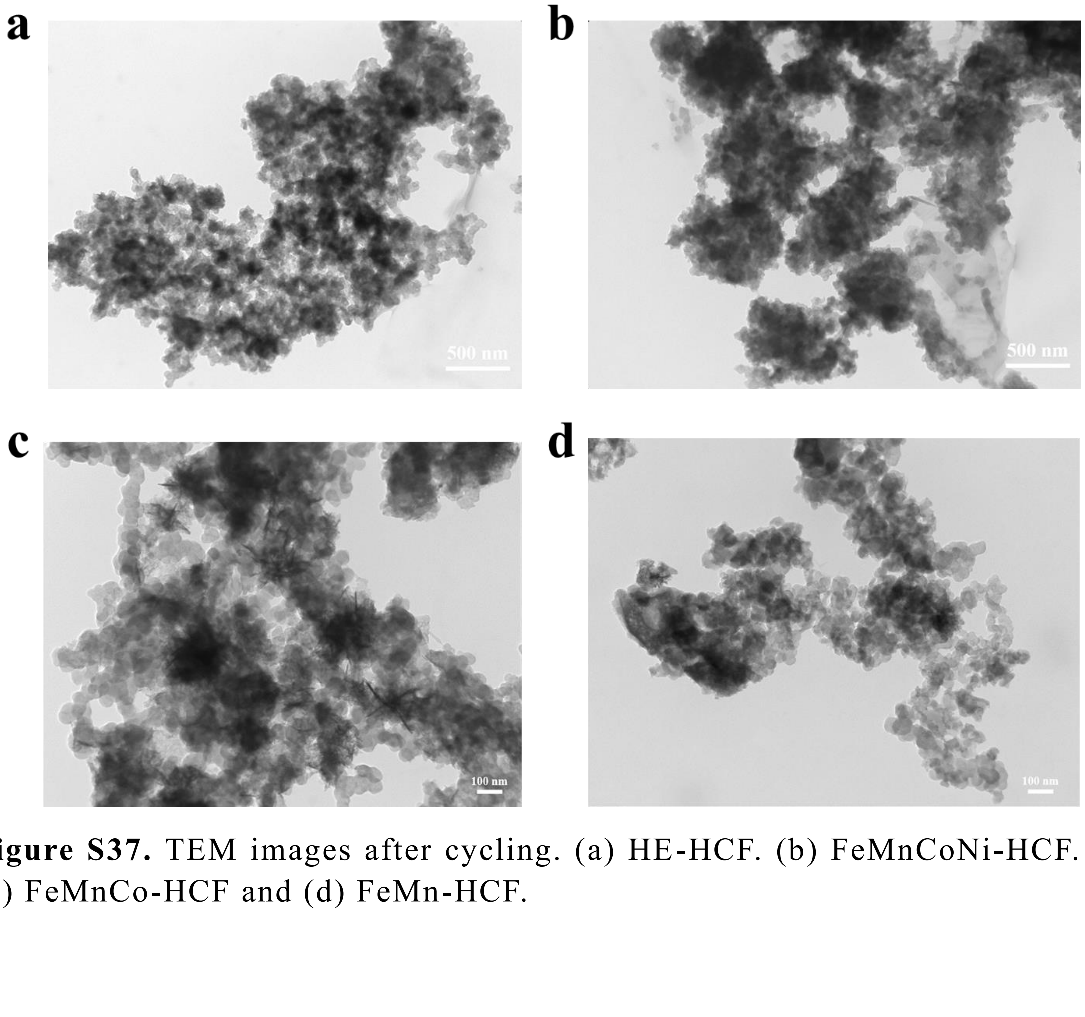

Figure S37. TEM images after cycling. (a) HE-HCF. (b) FeMnCoNi-HCF. (c) FeMnCo-HCF and (d) FeMn-HCF.

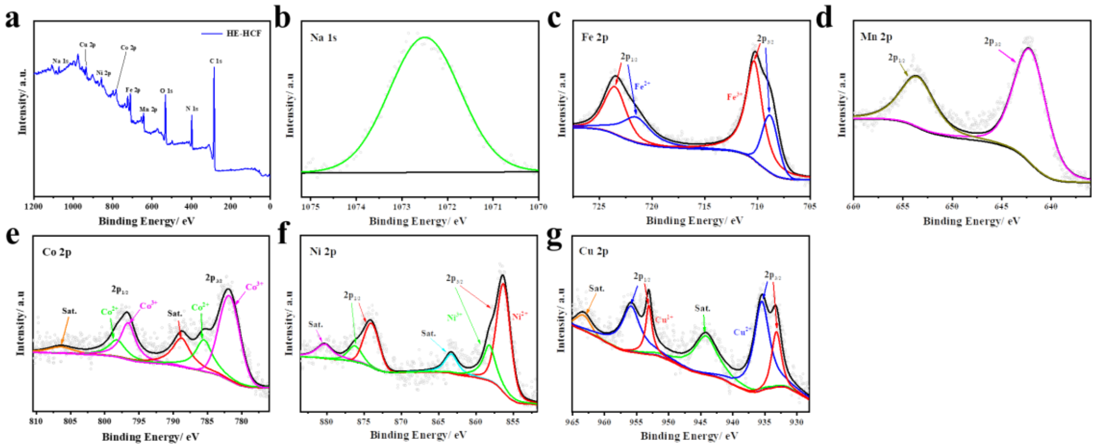

Figure S38. XPS survey spectrum of HE-HCF after cycling of (a) XPS survey spectra. (b) Na 1s. (c) Fe 2p. (d) Mn 2p. (e) Co 2p. (f) Ni 2p and (g) Cu 2p.

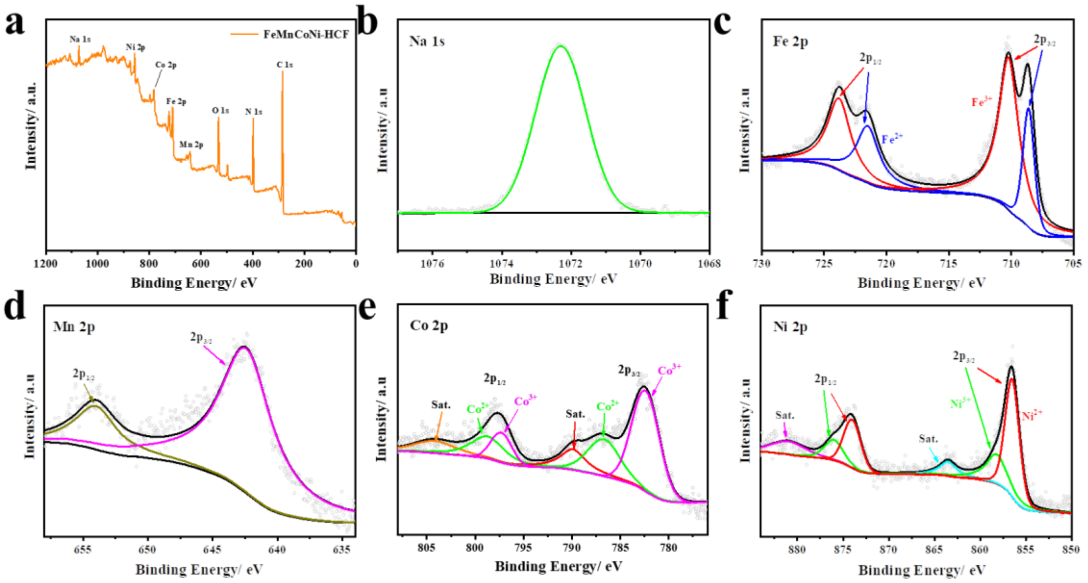

Figure S39. XPS survey spectrum of FeMnCoNi-HCF after cycling of (a) XPS survey spectra. (b) Na 1s. (c) Fe 2p. (d) Mn 2p. (e) Co 2p and (f) Ni 2p.

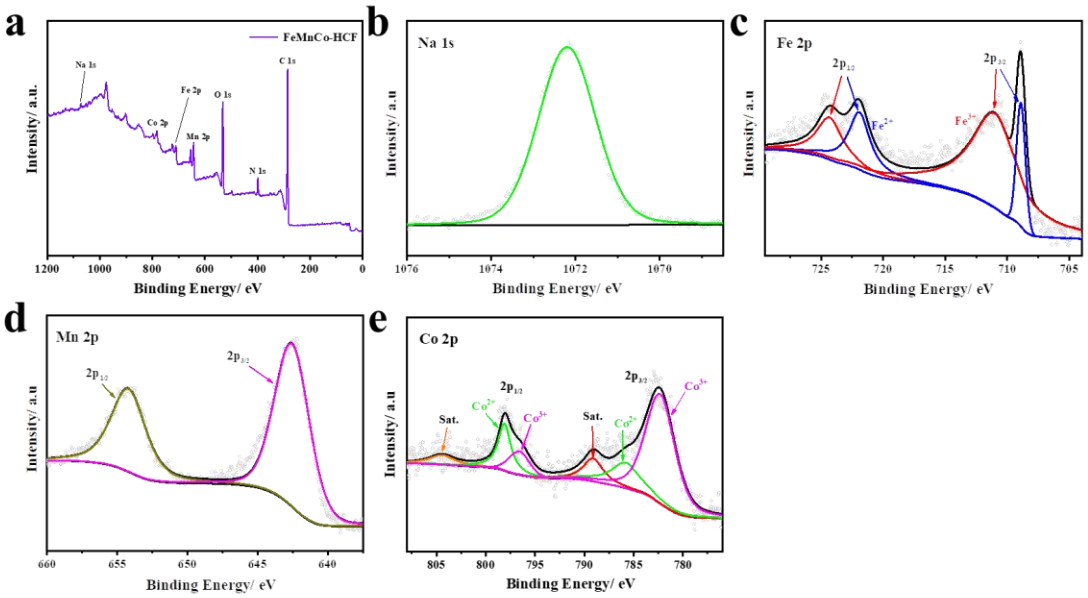

Figure S40. XPS survey spectrum of FeMnCo-HCF after cycling of (a) XPS survey spectra. (b) Na 1s. (c) Fe 2p. (d) Mn 2p and (e) Co 2p.

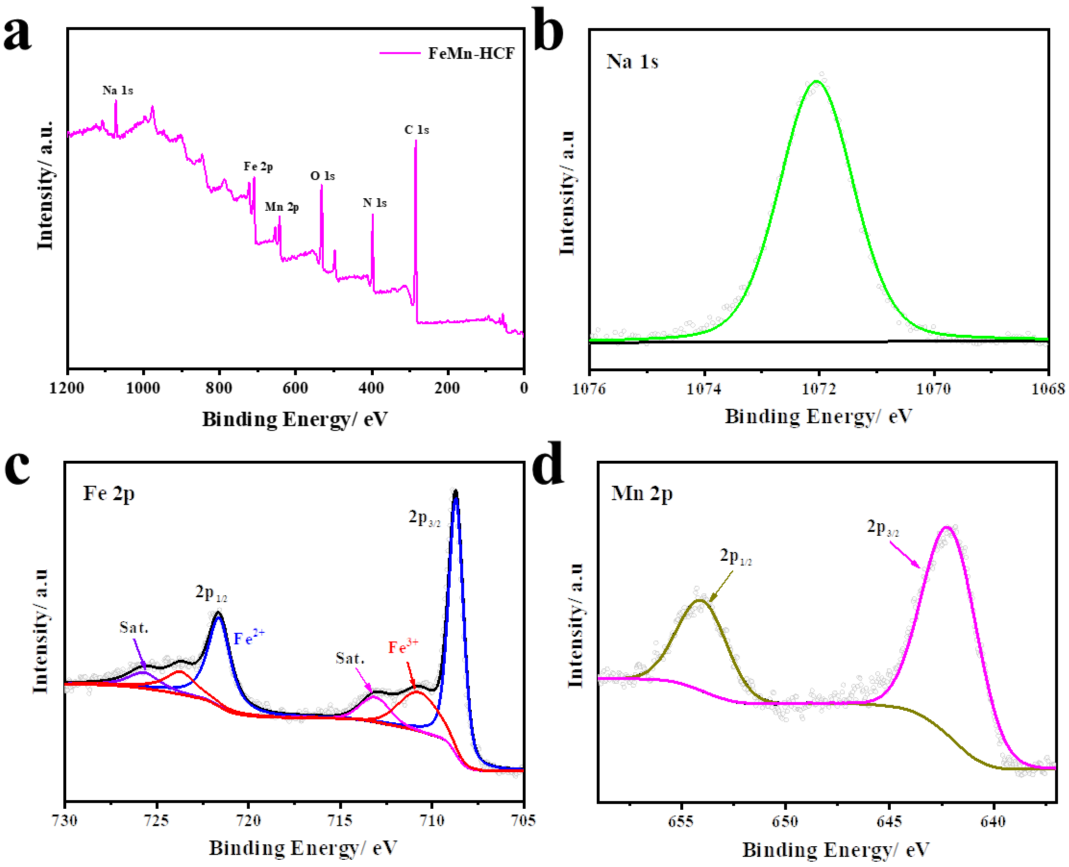

Figure S41. XPS survey spectrum of FeMn-HCF after cycling of (a) XPS survey spectra. (b) Na 1s. (c) Fe 2p and (d) Mn 2p.

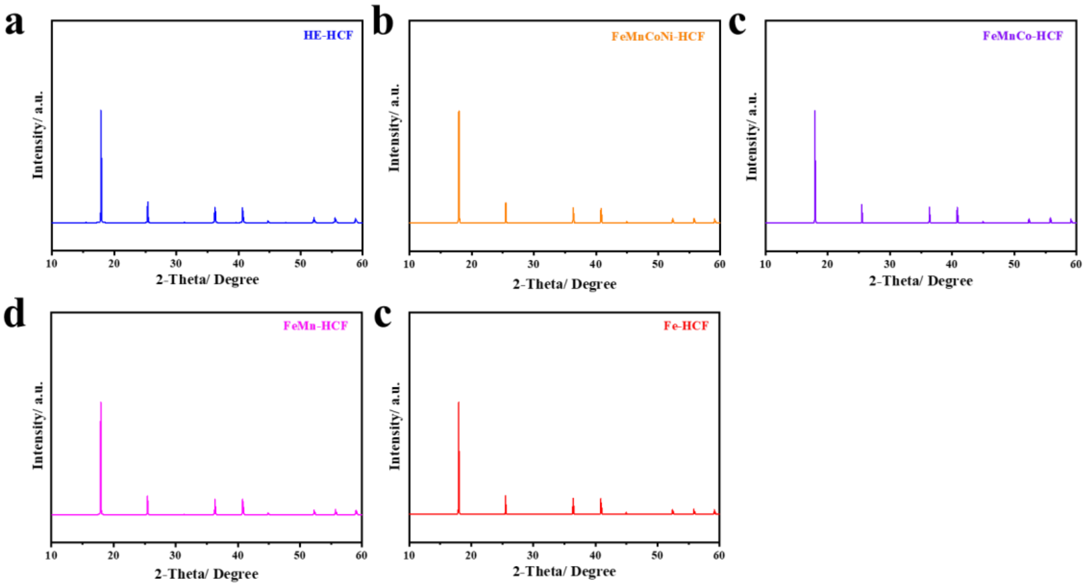

Figure S42. The calculated XRD patterns of different HCFs.

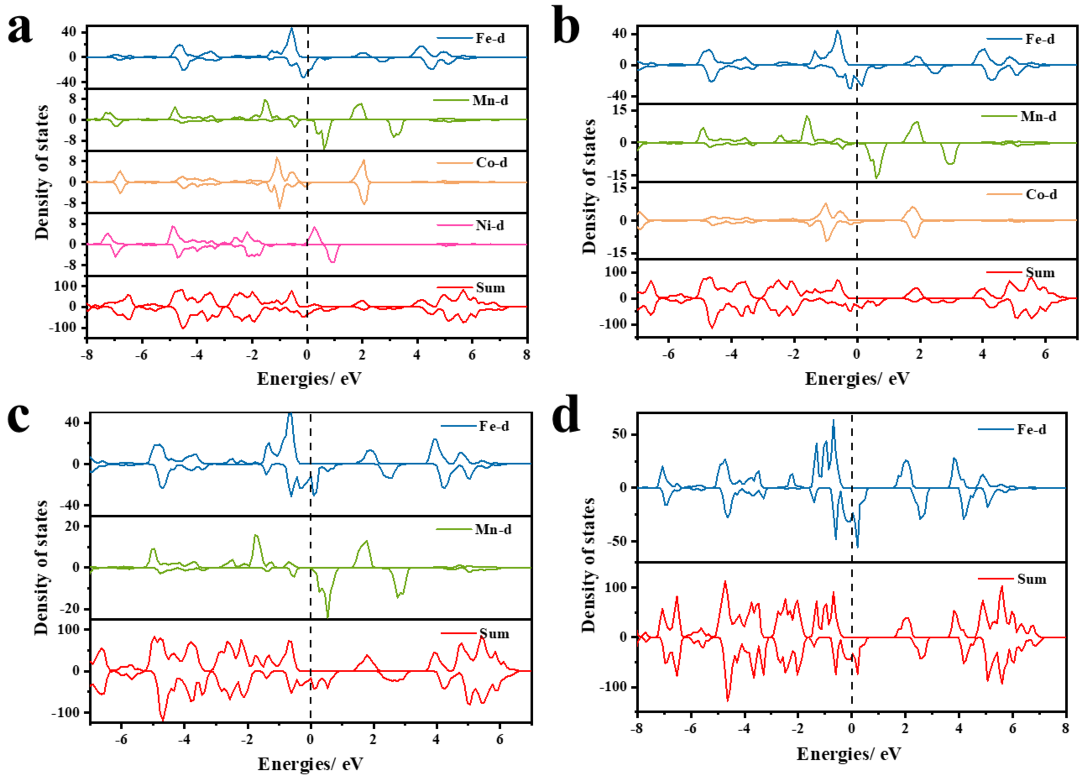

Figure S43. The TDOS and PDOS of metals in (a) FeMnCoNi-HCF. (b) FeMnCo-HCF. (c) FeMn-HCF and (d) Fe-HCF.

Table S1. Detailed structural information on the HE-HCF.

HE-HCF. Cubic, space group Fm-3m, $a=b=c=10.25012$Å, $\alpha=\beta=\gamma=90.0000°$, $V=1076.929$Å^3, $R_{wp}=7.45\%$ and $R_p=5.59\%$.

Atom	x	y	z	Occupation	U	Site
C	0.21748	0	0	1	0.023	24e
N	0.33641	0	0	1	0.032	24e
Na	0.25	0.25	0.25	0.167	0.01	8c
Fe	0	0	0	0.204	0.024	4b
Mn	0	0	0	0.194	0.024	4a
Co	0	0	0	0.231	0.024	4a
Ni	0	0	0	0.186	0.024	4a
Cu	0	0	0	0.186	0.024	4a
Fe	0.5	0	0	1	0.004	4a

Table S2. Mass fractions of metallic elements in the as-synthesized HCFs determined by ICP-OES.

Sample	Element	Mass fraction/ %
HE-HCF	Na	11.17
	Fe	7.08
	Mn	2.41
	Co	2.10
	Ni	2.51
	Cu	2.29
FeMnCoNi-HCF	Na	12.51
	Fe	9.59
	Mn	3.91
	Co	2.93
	Ni	3.24
FeMnCo-HCF	Na	14.14
	Fe	4.50
	Mn	2.31
	Co	2.30
FeMn-HCF	Na	12.89
	Fe	6.92
	Mn	5.55
Fe-HCF	Na	13.68
	Fe	21.49

Table S3. Mass fractions of nitrogen and carbon elements in the as-synthesize HCFs determined by EA.

Sample	Element	Mass fraction/ %
	C	17.33
HE-HCF	N	18.62
	H	1.703
	C	18.14
FeMnCoNi-HCF	N	19.32
	H	1.600
	C	18.33
FeMnCo-HCF	N	20.38
	H	1.166
	C	18.96
FeMn-HCF	N	20.68
	H	1.267
	C	18.21
Fe-HCF	N	18.23
	H	1.951

Table S4. The chemical formulas of the as-prepared samples with vacancies.

Sample	Chemical formula
HE-HCF	$Na_{1.117}Fe_{0.182}Mn_{0.216}Co_{0.183}Ni_{0.219}Cu_{0.2}[Fe(CN)_6]_{0.768}\square_{0.232}\cdot 2.15H_2O$
FeMnCoNi-HCF	$Na_{1.251}Fe_{0.211}Mn_{0.283}Co_{0.250}Ni_{0.256}[Fe(CN)_6]_{0.789}\square_{0.211}\square\cdot 2.08H_2O$
FeMnCo-HCF	$Na_{1.411}Fe_{0.315}Mn_{0.343}Co_{0.342}$ $[Fe(CN)_6]_{0.792}\square_{0.208}\square\cdot 2.06H_2O$
FeMn-HCF	$Na_{1.289}Fe_{0.469}Mn_{0.531}$ $[Fe(CN)_6]_{0.774}\square_{0.226}\square\cdot 2.05H_2O$
Fe-HCF	$Na_{1.368}Fe$ $[Fe(CN)_6]_{0.794}\square_{0.206}\square\cdot 2.25H_2O$

Table S5. Element content of Samples in NaCl solution after cycling.

Sample \ Element/ppm	Fe	Mn	Co	Ni	Cu
HE-HCF	0.0019	0.0994	0.0829	0.0509	0.0052
FeMnCoNi-HCF	0.0062	0.4164	0.3232	0.2317	0
FeMnCo-HCF	0.2523	0.4624	0.4642	0	0
FeMn-HCF	1.699	1.339	0	0	0

Table S6. Element content of Samples in NaCl solution after cycling.

Materials	Voltage /V	Current density /mA g^{-1}	NaCl /mg L^{-1}	Desalination capacity /mg g^{-1}	Cycle number	Capacity retention /%	Ref.
NiHCF/CNT	1.2	None	500	29.1	30	62	[3]
NaFeHCF@CNT	1.2	None	3000	52.96	50	89.82	[4]
NCP/CNT	1.2	None	500	41.25	50	92.1	[5]
NiHCF/MXene	1.2	None	500	26.3	30	84	[6]
NiHCF/rGO	0-1.2	None	500	31.8	100	11	[7]
MXene/CuHCF	1.4	None	500	69.2	50	94.2	[8]
NiCoFe-PBA	1.2	None	500	116	40	89.6	[9]
HCl-NC	1.2	None	1000	103.2	45	93.5	[10]
h-$Co(OH)_2$	1.4	100	584	3.3	100	90.2	[11]
Ti-15-800	1.2	None	584	62.8	20	91	[12]
NTMP/C	1.2	None	2000	72.2	100	90.6	[13]
FeHCF@3DNC	1.2	None	5850	65.5	30	92.6	[14]
C-MBPAN	1.2	None	1000	74.27	None	None	[15]
3D-EPD-Mxene	1.5	None	500	59	None	None	[16]
HCNB	1.4	None	500	32.3	50	95.4	[17]
$W_{18}O_{49}$@C	1.2	None	500	25.75	200	90	[18]
TFPDQGO	1.6	None	1000	58.4	50	84.5	[19]
$MoSe_2$/MCHS	1.2	None	500	45.25	25	98	[20]
FeOOH	1.2	None	500	35.12	50	98	[21]
NP-EHPC	1.2	None	500	24.14	150	73.5	[22]
ZTO-II	1.8	None	500	45	None	None	[23]
MoS_2/S,N-pC	1.4	None	750	47.9	20	96.7	[24]

2D $Ti_3C_2T_x$ Mxene	1.4	None	3000	39.7	50	75.8	[25]
$MnMoO_4$@*g* -C_3N_4/CNT	1.2	None	1000	43.6	10	91	[26]
2D-Fe_3O_4/C-450	1.2	None	500	28.5	None	None	[27]
Co_3O_4-rGO	1.6	None	250	18.63	100	46.2	[28]
MnO@C	1.2	None	500	31.3	15	95	[29]
MoS_2@CNT-CS	1.2	None	500	25.35	30	98.8	[30]
Co@CP	1.2	None	500	47.8	30	77	[31]
MoS_2@NCS-800	1.4	None	2000	59.9	100	63	[32]
TiO_2/Ti_3C_2	1.2	15	500	75.62	200	71	[33]
MnO_2/RGO	1.2	None	500	52	10	97	[34]
$MnCo_2O_4$	1.2	None	500	88.66	None	None	[35]
NJUST	1.4	None	250	15.59	8	89.1	[36]
NiHCF@rGO	1.2	None	4000	80.2	None	None	[37]
MXene/CNT	1.2	None	500	34.5	40	89	[38]
M-NTO/rGO	1.4	30	None	57.57	100	87.3	[39]
CS500	1.2	None	500	14.5	50	87	[40]
MoS_2@MXene	1.2	None	500	35.6	40	96	[41]
L-S-$Ti_3C_2T_x$	None	30	292	72	50	90	[42]
HE-HCF	1.2	None	500	77.24	350	97	This work

References

[1] J. W. Yeh, S. K. Chen, S. J. Lin, J. Y. Gan, T. S. Chin, T. T. Shun, C. H. Tsau, S. Y. Chang, *Advanced Engineering Materials* **2004**, 6, 299.

[2] C. H. Hu, Y. C. Chen, P. J. Yu, K. Y. Fung, Y. C. Hsueh, P. K. Liaw, J. W. Yeh, A. Hu, *Applied Physics Letters* **2019**, 115, 264103.

[3] L. Xu, Z. Ding, Y. Chen, X. Xu, Y. Liu, J. Li, T. Lu, L. Pan, *Journal of Colloid and Interface Science* **2023**, 630, 372.

[4] W. Zhang, X. Wei, X. Zhang, S. Huo, A. Gong, X. Mo, K. Li, *Separation and Purification Technology* **2022**, 287, 120483.

[5] J. Guo, Y. Wang, Y. Cai, H. Zhang, Y. Li, D. Liu, *Desalination* **2022**, 528, 115622.

[6] Z. Chen, Z. Ding, Y. Chen, X. Xu, Y. Liu, T. Lu, L. Pan, *Chemical Engineering Journal* **2023**, 452, 139451.

[7] Z. Ding, X. Xu, Y. Li, K. Wang, T. Lu, L. Pan, *Desalination* **2019**, 468, 114078.

[8] S. Wang, Z. Li, G. Wang, Y. Wang, Z. Ling, C. Li, *ACS Nano* **2022**, 16, 1239.

[9] X. Tu, Y. Liu, K. Wang, Z. Ding, X. Xu, T. Lu, L. Pan, *Journal of Colloid and Interface Science* **2023**, 642, 680.

[10] M. Liang, N. Liu, X. Zhang, Y. Xiao, J. Yang, F. Yu, J. Ma, *Advanced Functional Materials* **2022**, 32.

[11] Y. Xiong, F. Yu, S. Arnold, L. Wang, V. Presser, Y. Ren, J. Ma, *Research*, 2021.

[12] S. Cao, Y. Li, Y. Tang, Y. Sun, W. Li, X. Guo, F. Yang, G. Zhang, H. Zhou, Z. Liu, Q. Li, M. Shakouri, H. Pang, *Advanced Materials* **2023**, 35.

[13] S. Wang, G. Wang, C. He, N. Gao, B. Lu, L. Zhao, J. Weng, S. Zeng, C. Li, *Journal of Materials Chemistry A* **2021**, 9, 6898.

[14] A. Gong, Y. Zhao, M. He, B. Liang, K. Li, *Desalination* **2021**, 505, 114997.

[15] X. Cui, Z. Zhu, M. Song, M. Li, J. Qi, Y. Zhou, Y. Yang, J. Li, *Carbon* **2024**, 218, 118761.

[16] S. Lei, T. Yang, C. Tian, T. Yan, X. Li, W. Song, W. Liu, L. Yan, Y. Zhao, *Desalination* **2024**, 574, 117248.

[17] K. Li, S. Zhu, S. Zhao, M. Gong, X. Zhao, J. Liang, J. Gan, Y. Huang, M. Zhao, D. Zhuang, Q. Gong, *Journal of Materials Chemistry A* **2024**, 12, 899.

[18] W. Xing, K. Luo, J. Liang, C. Su, W. Tang, *Chemical Engineering Journal* **2023**, 477, 147268.

[19] L. Xu, Y. Liu, Z. Ding, X. Xu, X. Liu, Z. Gong, J. Li, T. Lu, L. Pan, *Small* **2023**, n/a, 2307843.

[20] J. Du, W. Xing, J. Yu, J. Feng, L. Tang, W. Tang, *Water Research* **2023**, 235, 119831.

[21] J. Zhao, B. Wu, X. Huang, Y. Sun, Z. Zhao, M. Ye, X. Wen, *Advanced Science* **2022**, 9, 2201678.

[22] H. Zhang, C. Wang, W. Zhang, M. Zhang, J. Qi, J. Qian, X. Sun, B. Yuliarto, J. Na, T. Park, H. G. A. Gomaa, Y. V. Kaneti, J. W. Yi, Y. Yamauchi, J. Li, *Journal of Materials Chemistry A* **2021**, 9, 12807.

[23] H. H. Kyaw, M. T. Z. Myint, K. Al-Yahmadi, M. Al-Abri, *Separation and Purification Technology* **2024**, 330, 125436.

[24] H. Sharifpour, F. Hekmat, S. Shahrokhian, *ACS Applied Materials & Interfaces* **2023**, 15, 42568.

[25]T. K. A. Nguyen, N. T. N. Anh, M. D. Nguyen, V. T. Nguyen, R.-a. Doong, *Separation and Purification Technology* **2023**, 327, 124934.
[26]V. M. Rangaraj, J.-I. Yoo, J.-K. Song, V. Mittal, *Separation and Purification Technology* **2023**, 317, 123898.
[27]L. Chen, X. Xu, L. Wan, G. Zhu, Y. Li, T. Lu, M. D. Albaqami, L. Pan, Y. Yamauchi, *Materials Chemistry Frontiers* **2021**, 5, 3480.
[28]G. Divyapriya, K. K. Vijayakumar, I. Nambi, *Desalination* **2019**, 451, 102.
[29]Y. Xu, S. Xiang, H. Mao, H. Zhou, G. Wang, H. Zhang, H. Zhao, *Nano Research* **2021**, 14, 4878.
[30]Y. Cai, W. Zhang, R. Fang, D. Zhao, Y. Wang, J. Wang, *Desalination* **2021**, 520, 115325.
[31]Z. Zhang, H. Li, *Chemical Engineering Journal* **2022**, 447, 137438.
[32]T. K. A. Nguyen, T.-H. Wang, R.-a. Doong, *Desalination* **2022**, 540, 115979.
[33]N. Liu, L. Yu, B. Liu, F. Yu, L. Li, Y. Xiao, J. Yang, J. Ma, *Advanced Science* **2023**, 10, 2204041.
[34]F. Ahmed, A. Umar, S. Kumar, N. M. Shaalan, N. Arshi, M. G. Alam, P. M. Z. Hasan, S. M. Ramay, R. Khan, A. Aljaafari, A. Alshoaibi, *Advanced Composites and Hybrid Materials* **2022**, 6, 19.
[35]H. Li, G. Peng, W. Wang, Z. Zhang, *Materials Letters* **2022**, 326, 132970.
[36]Z. Cao, S. Hu, Q. Yang, J. Yu, Y. Pan, J. Zuo, H. Song, Z. Ye, S. Zhang, *Chemical Engineering Journal* **2022**, 450, 138126.
[37]D. V. Cuong, C.-H. Hou, *Separation and Purification Technology* **2022**, 295, 121351.
[38]Y. Cai, L. Zhang, R. Fang, Y. Wang, J. Wang, *Separation and Purification Technology* **2022**, 292, 121019.
[39]X. Shen, L. Li, Y. Xiong, F. Yu, J. Ma, *Journal of Materials Chemistry A* **2022**, 10, 10192.
[40]D. Deng, M. K. Luhasile, H. Li, Q. Pan, F. Zheng, Y. Wang, *Desalination* **2022**, 531, 115685.
[41]Y. Cai, Y. Wang, L. Zhang, R. Fang, J. Wang, *ACS Applied Materials & Interfaces* **2022**, 14, 2833.
[42]X. Shen, Y. Xiong, R. Hai, F. Yu, J. Ma, *Environmental Science & Technology* **2020**, 54, 4554.
